# Visualising the cytoskeletal machinery in neuronal growth cones using cryo-electron tomography

**DOI:** 10.1242/jcs.259234

**Published:** 2022-04-08

**Authors:** Joseph Atherton, Melissa Stouffer, Fiona Francis, Carolyn A. Moores

**Affiliations:** 1 Randall Centre for Cell and Molecular Biophysics, King's College, London SE1 1YR, UK; 2 Institute of Structural and Molecular Biology, Birkbeck, University of London, London WC1E 7HX, UK; 3 INSERM UMR-S 1270, 17 Rue du Fer à Moulin, 75005 Paris, France; 4 Sorbonne University UMR-S 1270, 4 Place Jussieu, 75005 Paris, France; 5 Institut du Fer à Moulin, 17 Rue du Fer à Moulin, 75005 Paris, France; 6 Institute of Science and Technology Austria, Am campus 1, 3400 Klosterneuberg, Austria

**Keywords:** Neuron, Growth cone, Cytoskeleton, Actin, Microtubule, Doublecortin

## Abstract

Neurons extend axons to form the complex circuitry of the mature brain. This depends on the coordinated response and continuous remodelling of the microtubule and F-actin networks in the axonal growth cone. Growth cone architecture remains poorly understood at nanoscales. We therefore investigated mouse hippocampal neuron growth cones using cryo-electron tomography to directly visualise their three-dimensional subcellular architecture with molecular detail. Our data showed that the hexagonal arrays of actin bundles that form filopodia penetrate and terminate deep within the growth cone interior. We directly observed the modulation of these and other growth cone actin bundles by alteration of individual F-actin helical structures. Microtubules with blunt, slightly flared or gently curved ends predominated in the growth cone, frequently contained lumenal particles and exhibited lattice defects. Investigation of the effect of absence of doublecortin, a neurodevelopmental cytoskeleton regulator, on growth cone cytoskeleton showed no major anomalies in overall growth cone organisation or in F-actin subpopulations. However, our data suggested that microtubules sustained more structural defects, highlighting the importance of microtubule integrity during growth cone migration.

## Abstract (precis)

**Summary:** Cryo-electron tomographic reconstruction of neuronal growth cone subdomains reveals distinctive F-actin and microtubule cytoskeleton architectures and modulation at molecular detail.

## INTRODUCTION

During development, neurons first form primary extensions that direct cell migration, and then extend axons to form synapses. These pathfinding processes are directed by the axonal growth cone in response to guidance cues and driven by an integrated network of microtubules (MT) and actin filaments (F-actin). Growth cone malfunction causes aberrant axonal pathfinding and incorrect wiring of the nervous system, and is the basis of a number of neurodevelopmental disorders including a range of intellectual disability conditions, autism and schizophrenia (Bakos et al., 2015; Kim and Kim, 2020; Stoeckli, 2018). For example, mutations in the protein doublecortin (DCX in humans, Dcx in mouse), which is a regulator of both growth cone F-actin and MT organisation (Deuel et al., 2006; Friocourt et al., 2003; Fu et al., 2013; Jean et al., 2012; Tint et al., 2009), cause neurodevelopmental lissencephaly (Wynshaw-Boris et al., 2010). Understanding the organisation and regulation of the neuronal growth cone cytoskeleton can thus shed light on the molecular basis of numerous diseases (Roig-Puiggros et al., 2020).

Distinct growth cone regions have been characterised based on their appearance, organelle distribution and cytoskeletal composition (Geraldo and Gordon-Weeks, 2009) (Fig. S1A,B). The axon shaft that feeds into the growth cone is filled with parallel, tightly bundled MTs – large organelles such as mitochondria are common and F-actin is relatively sparse (Dent et al., 2011). The axon connects with the central domain (C-domain), which includes the ‘axon wrist’; like the axon, the C-domain contains large organelles and MTs, although C-domain MTs are more dispersed and dynamic (Blanquie and Bradke, 2018). The front region of the growth cone – the peripheral domain (P-domain) – is flat and organelle free. It consists of protrusions called filopodia, containing F-actin bundles, together with the lamellipodium, containing the branched F-actin network. The P-domain is separated from the C-domain by the transition zone (T-zone), which, as its name suggests, is a region of transition between the F-actin-dominated outer regions of the growth cone and the MT-rich axon-proximal regions. Contractile F-actin arcs formed by non-muscle myosin II run parallel to the front of the growth cone in the T-zone and help drive cellular motility (Burnette et al., 2008, 2007; Schaefer et al., 2002; Zhang et al., 2003). MTs only rarely grow from the T-zone into the P-domain and are often guided by F-actin bundles (Gordon-Weeks, 1991; Sabry et al., 1991; Schaefer et al., 2002, 2008). Furthermore, MTs that are found in the periphery are dynamic and unstable – exploratory MTs that are linked to F-actin retrograde flow often buckle and break in the T-zone (Schaefer et al., 2002, 2008; Waterman-Storer and Salmon, 1997).

There are a number of open questions concerning the molecular basis for growth cone cytoskeleton regulation and coordination. The organisation of non-filopodia P-domain F-actin and its connectivity and communication with the T-zone are not well understood. In particular, the mechanism by which filopodial F-actin bundles are disassembled and the origin of actin arcs in the T-zone are not clear. In addition, it is not known whether different growth cone MT populations have distinct structural signatures, and, although the involvement of a number of MT–actin crosslinkers has been proposed (Geraldo and Gordon-Weeks, 2009; Lowery and Van Vactor, 2009), how peripheral MTs in growth cones associate with different arrays of F-actin is currently unclear. Nanoscale insights into neuronal growth cone organisation can provide key insights to address some of these outstanding questions.

Cryo-electron tomography (cryo-ET) provides a window into the growth cone interior by enabling visualisation of intact frozen-hydrated cellular samples, and three-dimensional (3D) reconstruction enables ultrastructural characterisation of molecular components *in situ* (Turk and Baumeister, 2020). We therefore used cryo-ET and machine-learning-based segmentation and visualisation to investigate the complex molecular organisation of sub-compartments within mouse hippocampal neuron growth cones and the part of the axon that leads in to them. We focused particularly on their underlying cytoskeletal architectures that are vital to axon pathfinding. We also compared the growth cone ultrastructure in mouse wild-type neurons with those of Dcx knockout neurons, to evaluate the effect of Dcx loss on the cytoskeleton.

## RESULTS

### Cryo-ET of primary hippocampal neuron growth cones

We chose mouse primary hippocampal neurons as a model system because the biology of their growth cone formation and function is well studied, and because the Dcx knockout phenotype in mice manifests itself most prominently in a hippocampal malformation (Corbo et al., 2002; Kappeler et al., 2007). After 2–3 days of *in vitro* culture on laminin- and poly-D-lysine-coated coverslips, these cells exhibited several short minor dendritic processes, together with an extended axon tipped by a growth cone (Fig. S1A; reviewed in Banker, 2018). Dcx mostly colocalises with MTs towards the outer ends of processes and in the growth cone C-domain (Bielas et al., 2007), but less intense Dcx staining was also seen in the P-domain colocalised with actin (Fig. S1A,B) (Fu et al., 2013; Tint et al., 2009).

Mouse primary hippocampal neurons were therefore grown on laminin- and poly-D-lysine-treated holey carbon electron microscopy (EM) grids for 2–3 days. Grids were vitrified, growth cones identified at low magnification (Fig. S1C), tilt series collected and tomographic volumes calculated, allowing clear visualisation of the interior of the neuron (Fig. S1D, Movie 1).

### Visualisation of the axonal growth cone molecular landscape

**Fig. 1.**
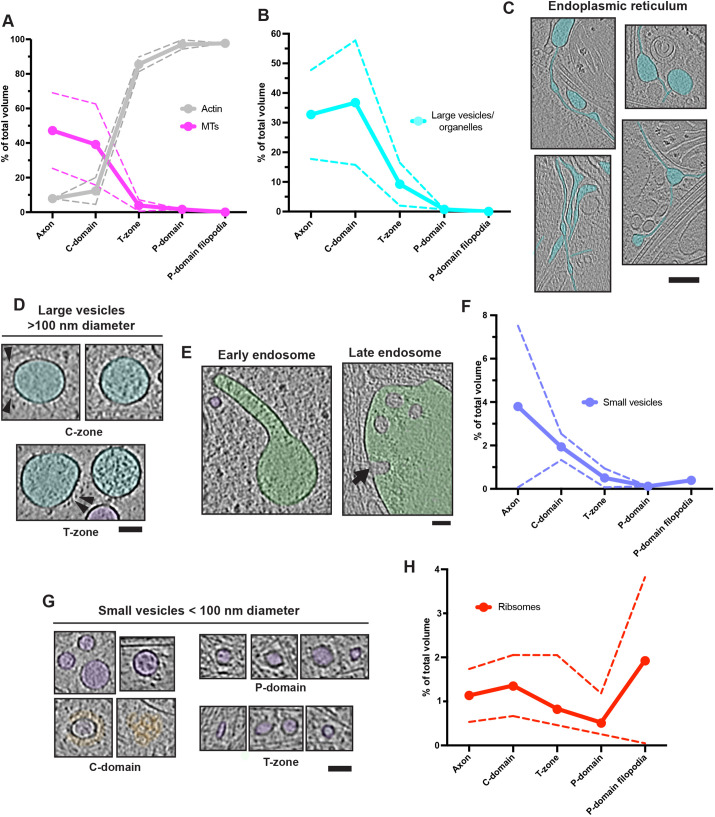
**Analysis of growth cone component distribution.** (A) Growth cone distribution of microtubules (MTs) and F-actin segmentation volumes. (B) Large vesicle and organelle (including mitochondria) segmentation volumes. (C) C-domain smooth ER, cyan false colour. (D) Large vesicles, false coloured in cyan. Arrowheads, embedded proteins. (E) C-domain endolysosomal components (left: early/sorting endosome; right: late endosome/multi-vesicular body), false coloured in green. Arrow indicates inward budding site. (F) Large vesicle and organelle segmentation volumes. (G) Small vesicles <100 nm diameter, false coloured in purple; clathrin coats, false coloured in orange. (H) Segmentation volume of ribosomes. Panels C–E and G show longitudinal views in tomograms (4× binned). Absolute segmentation volumes were measured in Chimera (Pettersen et al., 2004), and relative segmentation volume for each feature is expressed as a percentage of the total volume of all these features combined. Axon, *n*=2; C-domain, *n*=3; T-zone, *n*=3; P-domain, *n*=2; P-domain filopodia, *n*=2. Solid lines represent mean volumes, dashed lines indicate s.d. Scale bars: C, 100 nm; D,E,G, 60 nm.

We used neural-network-based approaches to perform semi-automated segmentation of the growth cone tomograms (Fig. S2), enabling 3D visualisation of their molecular organisation (Fig. S3). We also used the segmentation analysis to assess the molecular distribution of cellular components within the different growth cone regions (Fig. 1), consistent overall with previous observations (Geraldo and Gordon-Weeks, 2009). There was a sharp drop in MT density from the C-domain through the T-zone into the P-domain, which was the inverse of F-actin distribution, where F-actin arrays dominated the growth cone periphery (Fig. 1A). Larger membrane-bound organelles were relatively common in the C-domain (Fig. 1B), including endoplasmic reticulum (ER) networks (Fig. 1C), large vesicles (>100 nm diameter, Fig. 1D) and endolysosomal system components (Fig. 1E). In the P-domain, these cellular components were very rare and were perhaps excluded because of their physical dimensions. The distribution of smaller (<100 nm diameter) vesicles was more variable among different growth cones (Fig. 1F). Whereas a variety of small vesicles were observed in the P-domain and even in filopodia, those with clathrin-coats were only observed in the C-domain (Fig. 1G). Individual ribosome and polysome distributions remained relatively constant throughout the growth cone, except in the filopodia, where they were more abundant, especially compared to the rest of the P-domain (Fig. 1H).

### Pseudo-crystalline organisation of neuronal filopodia actin bundles

**Fig. 2.**
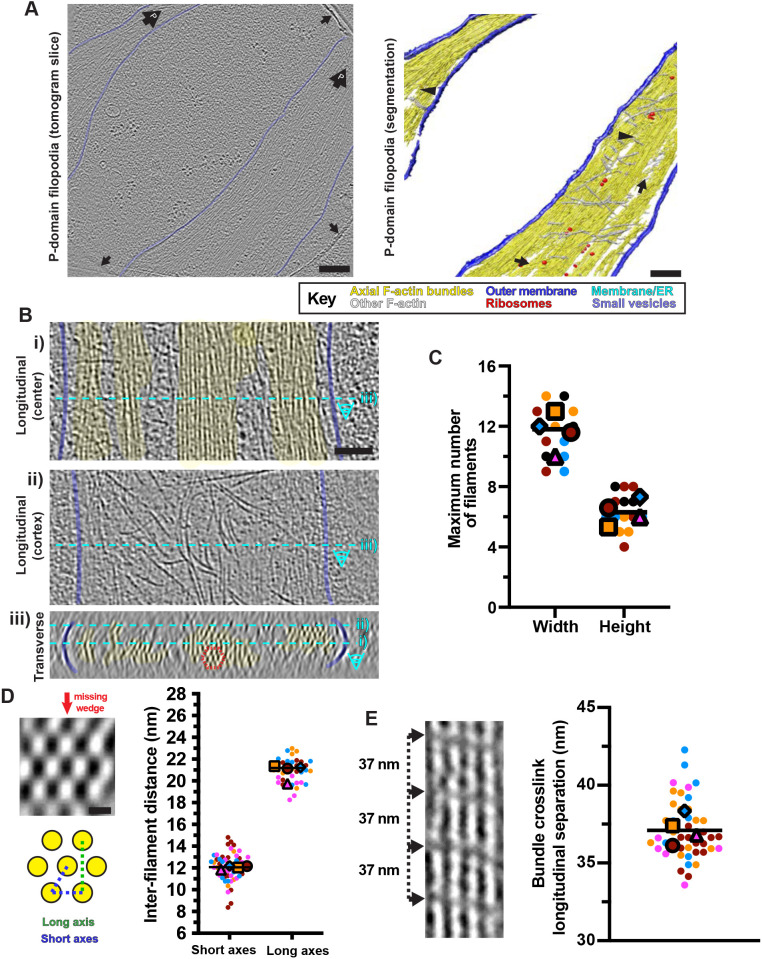
**Ultrastructure of filopodial F-actin arrays.** (A) Central tomogram section (left, 4× binned), segmentation (right) of two filopodia. Left: large arrows labelled ‘P’ show position of cell front; smaller arrows show hole edges. Right: arrows indicate filaments that connect bundles; arrowheads indicate merging of bundles. Key indicates false colour scheme. (B) Tomogram sections (4× binned) of a large filopodium showing the (i) central region, (ii) cortex and (iii) cross-section (∼20 nm depth). Cyan dashed lines indicate relationship of panels. Dashed red hexagon indicates a hexagonal F-actin bundle unit. (C) Super-plot (Lord et al., 2020) of maximum width and height of filopodial bundles. Each data point represents a filopodial bundle; *n*=14 from 4 tomograms, each a different colour and shape; tomogram mean values are plotted in the same colour with larger shapes. Overall width, 11.6±1.7 nm; height, 6.4±1.2 nm; values represent the mean±s.d.; the line represents the overall median. (D) Top left: transverse view through a filopodial bundle (∼20 nm depth), showing hexagonal arrangement, equivalent but in a different bundle compared with panel B; red arrow shows missing-wedge direction. Bottom left: schematic of a hexagonal unit of 7 filaments; blue and green dashed lines indicate short and long inter-filament distances, respectively. Right: super-plot of inter-filament distances in filopodial bundles. Each data point represents a distinct, adjacent filament pair (short axes, *n*=60 from 4 tomograms; long axis, *n*=36 from 4 tomograms; each tomogram shown as a different colour; tomogram mean values are plotted in the same colour with larger shapes. Line indicates the overall mean (short axes, 12.1±1.2 nm; long axes, 20.9±1.2 nm; values represent the mean±s.d.). (E) Left: a single crosslinked actin sheet in a filopodial bundle (4× binned) showing ∼37 nm crosslink separation, indicated with arrows. Right: Super-plot of longitudinal crosslink separation in P-domain F-actin bundles. Each data point represents an individual pair of crosslinks along the F-actin longitudinal axis (*n*=44 from 4 tomograms, each tomogram shown as a different colour). Tomogram mean values are plotted in the same colour with larger shapes. Line indicates the overall mean (37.1±1.9 nm; mean±s.d.). Scale bars: A, 200 nm; B, 100 nm; D, 10 nm.

The molecular detail visible in our tomograms allowed us to characterise the diverse manifestations of the actin cytoskeleton that dominate outer growth cone regions. At the very front of the growth cones, several filopodial regions were typically captured in each tomogram (Fig. 2A; Fig. S3D), the dominant feature of which was tight bundles of F-actin filaments (Fig. 2B). Surrounding the bundles, individual actin filaments lay beneath the membrane, some running roughly perpendicular to the filopodial axis (Fig. 2A,B), and some of which incorporated into the bundles. Smaller filopodial extensions (< 400 nm diameter) contained a single F-actin bundle, whereas larger filopodia contained two to three discrete bundles, which exchanged filaments or coalesced (Fig. 2B). Other non-cortical, non-bundled actin was also found flanked by bundles, or between bundles and the membrane (Fig. 2B).

Filaments within all the filopodia were arranged in a characteristic 2D hexagonal pseudo-crystalline array (Fig. 2B) (Kureishy et al., 2002), although this arrangement was not strictly maintained – the positions of individual filaments meandered within the larger bundles, and bundle thickness varied (Movie 2). We observed bundles of 4–8 filaments in height and 9–14 filaments in width (Fig. 2C), with an inter-filament spacing of ∼12 nm (Fig. 2D). The bundle cross-sections also showed the elongating effect of the tomogram's missing wedge on the otherwise approximately cylindrical actin filaments (Fig. 2D). Connecting densities between adjacent bundle filaments were also observed, spaced at ∼37 nm intervals along the diagonals of the hexagonal array (Fig. 2E).

**Fig. 3.**
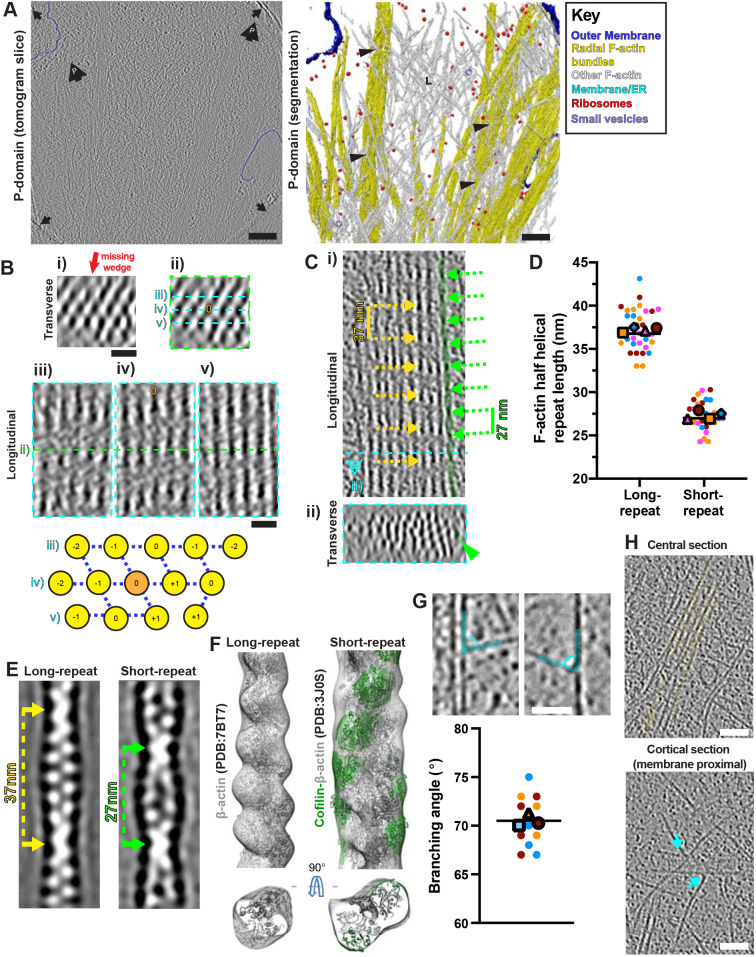
**Complexity and modulation of F-actin arrays in growth cone P-domains.** (A) Central tomogram section (4× binned, left), segmented rendering (right) of a P-domain region. Left: large black arrows containing a ‘P’, position of cell front; black arrows, carbon hole edges. Right: L, lamellipodial; black arrowheads, branched F-actin networks surrounding F-actin bundles. Key indicates false colouring. (B) (i) Transverse view (2× binned) through filopodial bundle (∼20 nm depth), showing the hexagonal arrangement; red arrow, missing-wedge direction. (ii) Same bundle and view; cyan dashed lines, longitudinal sections of panels. (iii–v) The centre of the transverse section in i and ii is indicated with a green dashed line in panels iii–v. A central filament is indicated, orange ‘0’ in panels ii and iv. Bottom: transverse schematic of filament arrangement, with the central ‘0’ filament in orange. Numbers indicate how longitudinal F-actin repeats shift by integers of 5.5 nm along the filament axis relative to the central filament. (C) (i) Longitudinal and (ii) transverse (∼20 nm depth) views of a large F-actin bundle in a 2x binned P-domain tomogram. Dashed cyan line in i illustrates position of transverse section in ii. ∼37 nm F-actin half helical repeat lengths indicated with yellow dashed arrows, rarer ∼27 nm F-actin half helical repeat lengths indicated with green dashed arrows. A short-repeat filament is false coloured in green and indicated in ii with a green arrowhead. (D) Super-plot of P-domain F-actin half helical repeat lengths. Individual points indicate single measurements along a filament axis, colours indicate different filaments. Mean values for each filament are shown in their respective colours with different larger shapes. Individual lines indicate overall median values. Repeat lengths for long-repeat filaments, 37.2±2.4 nm *n*=32, 4 filaments (3 tomograms); repeat lengths for short-repeat filaments; 27.3±1.6 nm, *n*=30, 4 filaments (3 tomograms). Values represent the mean±s.d. (E) Projections through subtomogram averages of long-repeat (left) and short-repeat (right) filaments showing half repeat distances. The sub-tomogram densities were calculated by averaging 1461 and 621 volumes for long-repeat and short-repeat filaments, respectively. Volumes were low-pass filtered to their estimated resolutions at Fourier shell correlation (FSC)=0.5 (Fig. S6). (F) Subtomogram averages of long-repeat filaments (left, mesh density) and short-repeat (right, mesh density) filaments with fitted F-actin (PDB: 7BT7) or F-actin–cofilin (PDB: 3J0S) models, respectively, noting that polarity of filament fitting is arbitrary and cannot be determined from the reconstructions. F-Actin and cofilin models are coloured in grey and green, respectively, with mesh density coloured to match the underlying models (within 10 Å). (G) P-domain tomogram sections (4× binned) illustrating F-actin branching from bundles (top left) and single filaments (top right), indicated with cyan false colouring. Bottom: Super-plot of P-domain branching angles. Each data point represents an individual branching structure (*n*=12 from 3 tomograms, shown as different colours). Mean values for each tomogram are shown in their respective colours with different larger shapes. Line indicates the overall mean (70.1±2.6°, mean±s.d.). (H) Longitudinal sections of a 2× binned P-domain tomogram through a central region showing radial F-actin bundles in false yellow colour (top) and the corresponding overlying cortical region (bottom), showing F-actin branching points at the cell cortex, indicated with cyan arrows. Scale bars: A, 200 nm; B, 20 nm; G, 40 nm; H, 50 nm.

This characteristic hexagonal filament organisation acts as a molecular signature to track filopodia-derived bundles into the P-domain cytoplasm. Our data show how they penetrate and integrate into the complex actin-rich environment of the P-domain (Fig. 3A; Fig. S3C). The filament arrangement was consistent between different bundles, both within the same filopodia and between different filopodia, and in bundles that extended into the P-domain (Figs 2 and 3; Fig. S4). Examination of bundle cross-sections showed that the helical repeats of neighbouring filaments were most often offset from each other by one subunit (Fig. 3B; Fig. S4D,E). This strongly suggests that bundling proteins are involved in forming and maintaining these ordered arrays. However, the variation in filament offset within the hexagonal arrays also means that even though sub-tomogram averaging could, in principle, reveal more structural details about bundle arrangement and allow missing wedge compensation, it would in fact result in averaging together of filaments that are not necessarily helically in phase. This would ultimately blur filament and crosslink density. Nevertheless, even without averaging, these data show distinctive and consistently ordered filament arrangements within these bundles.

### Modulation of F-actin arrays in growth cone P-domains

Strikingly, despite the conservation of the core structure of the filopodial bundles, a minority of filaments – either within the bundles, particularly at their periphery, or in separate filaments in the P-domain (Fig. 3C; Fig. S5A–C) – have a distinct appearance and a shorter half helical repeat length of ∼27 nm (Fig. 3D). There was no evidence of filaments with intermediate repeat lengths (Fig. 3D), and the ∼27 nm repeat filaments tended to be shorter and lacked regular crosslinks to adjacent filaments (Fig. 3C; Fig. S5). Furthermore, these short-repeat filaments generally meandered more within the bundle and often appeared to disrupt the regularity of the bundle with which they were associated (Fig. S5A,B). Some individual filaments clearly transitioned from one helical repeat to the other (Fig. S5C), whereas some single short-repeat filaments were found outside of bundles (Fig. S5D).

Sub-tomogram averages of the ∼37 nm and ∼27 nm half helical repeat filament populations (Fig. 3E; Fig. S5E) recapitulated the actin twists and helical repeats visible in 2D slices (Fig. 3C). Further, the ∼27 nm half helical repeat filament reconstruction suggested that one or more proteins are bound. Based on extensive prior knowledge of the role of cofilins and actin depolymerising factor (ADF) in actin cytoskeleton regulation (Gungabissoon and Bamburg, 2003) and the known structural twist imposed by cofilin/ADF on F-actin filaments (Galkin et al., 2011, 2001; Huehn et al., 2020; McGough et al., 1997; Tanaka et al., 2018), we rigid-body fitted F-actin–cofilin (PDB: 3J0S; Galkin et al., 2011) within this density (Fig. 3F; Movie 3). The known binding site of cofilin – longitudinally between actin subunits along F-actin – corresponded well qualitatively with the non-actin density in the reconstruction, although the resolution was not sufficient to distinguish either actin polarity or to determine the identity of the bound protein(s). Our ability to visualise this density nevertheless suggests high binding-protein occupancy, which, together with the observation of extended stretches of ∼27 nm half helical repeat filaments in the raw tomograms, is consistent with the reported cooperativity of cofilin binding *in vitro* (Huehn et al., 2018).

The P-domain also contained branched F-actin networks formed from a mixture of single filaments and small bundles between the filopodia-derived actin bundles (Fig. 3A). Branching originated from the sides of single filaments and, less frequently, from bundles, all at ∼70° angles (Fig. 3G), consistent with actin-related protein 2/3 (Arp2/3) complex activity (Dent et al., 2011). Although this branched network was distributed throughout the P-domain, the branch points themselves were primarily located close to the dorsal and ventral membranes (Fig. 3H).

### The mixed cytoskeletal economy of the T-zone

**Fig. 4.**
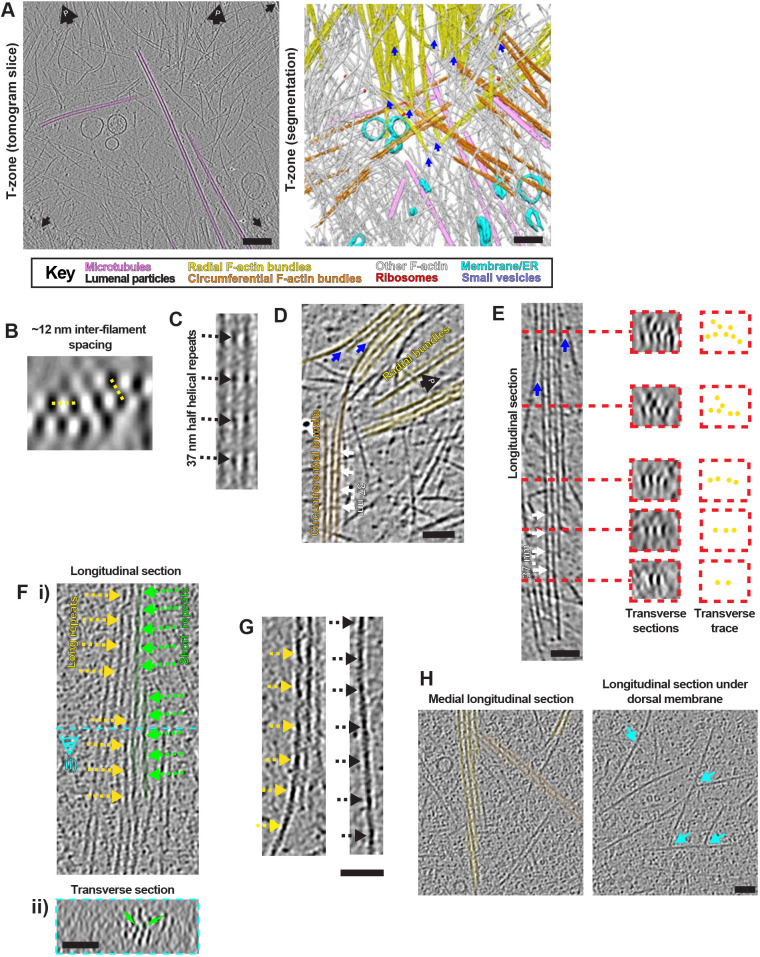
**Diverse F-actin arrays in neuronal growth cone T-zone.** (A) Central tomogram section (4× binned, left), segmented rendering (right) of T-zone. Left: large black arrows containing a ‘P’, position of cell front; black arrows, carbon hole edges. Key indicates false colouring. (B) Transverse section through radial F-actin bundle in T-zone tomogram (4× binned), showing hexagonal filament organisation; yellow dashed lines indicate ∼12 nm inter-filament distances. (C) Longitudinal section through radial F-actin hexagonal bundle in T-zone tomogram (2× binned), showing 37 nm half helical repeat lengths indicated by arrows. (D) Longitudinal section through tomogram (4× binned) of radial F-actin bundle taper and derived circumferential F-actin bundle, white arrows indicating ∼37 nm F-actin crosslinks. (E) Longitudinal (left) and transverse (centre, ∼20 nm depth) sections, indicated with red dashed lines, of tapering radial F-actin bundle in a T-zone tomogram (4× binned). Traced representation (right) illustrates bundle tapering. White arrows indicate 37 nm inter-crosslink spacing. (F) (i) Longitudinal and (ii) transverse views of tapering radial F-actin bundle in T-zone tomogram (2× binned). In i, distal and proximal are top and bottom, respectively. Dashed cyan line in i indicates the position of the transverse section in ii. ∼37 nm and ∼27 nm half helical repeat lengths are indicated with yellow and green dashed arrows, respectively. Short-repeat filament lengths are false-coloured in green and their transverse positions within the bundle indicated in ii with green arrows. (G) Longitudinal tomogram sections (2× binned) of radial F-actin bundle taper (left) and F-actin bundle-derived dislocated single filament (right), dashed arrows indicate long F-actin half helical repeat lengths. (H) Longitudinal sections of T-zone tomogram (2× binned, left), central region showing radial and circumferential F-actin bundles in false yellow and orange colours, respectively; corresponding overlying cortical region (right), showing F-actin branching points at cell cortex. Branching points are illustrated with cyan arrows. Blue arrows in A, D, E indicate points where filaments bend away from tapering radial F-actin bundles. Scale bars: A, 200 nm; D,E, 50 nm, F–H, 40 nm.

The T-zone cytoskeleton is characterised by (1) the termination of radially organised P-domain-derived F-actin bundles and (2) the termination of the majority of MTs originating from the C-domain. Filopodia-like F-actin bundles extended into the T-zone (Fig. 4A), orientated radially towards the front of the growth cone. Although these bundles contained fewer filaments (maximum height of approximately four filaments, maximum width of eight filaments), they exhibited the characteristic average inter-filament spacing, roughly hexagonal distribution, and ∼37 nm half helical repeat lengths seen in the P-domain (Fig. 4B,C). These bundles tended to taper within the T-zone, with sub-bundles and single filaments often bending off perpendicularly to the main bundle axis (Fig. 4A,D). However, even when filaments were only crosslinked in one dimension, they were regularly spaced and connected by crosslinking density at ∼37 nm intervals (Fig. 4D,E). As was also observed in the P-domain, a minority of filaments, typically shorter and at the periphery of the bundles, exhibited a ∼27 nm half helical repeat length (Fig. 4F; Fig. S5D).

Compared to the P-domain, the radial actin bundles were less dense in the T-zone, (Fig. 4A–C), and were interspersed among smaller bundles orientated parallel to the growth cone circumference (Fig. 4A; Fig. S3B). Additionally, numerous individual filaments ran parallel or perpendicular to the radial bundles (Fig. 4A; Fig. S3B). These distinct T-zone actin populations were often clearly derived from axial bundles (Fig. 4A,D–G), although their overall organisation was looser and apparently less precisely organised, particularly at the cell membrane. Circumferentially organised F-actin also ran roughly perpendicular to the periphery-orientated MTs, but did not appear to block their trajectory (Fig. 4A). Branched actin arrays consistent with Arp2/3 activity were also visible near the T-zone membrane (Fig. 4H). Thus, although tomograms of the growth cone T-zone showed that the F-actin network is overall less dense than the P-domain, they also clearly demonstrated that the actin cytoskeleton is organised in distinct populations.

### Growth cone microtubule architecture and interactions with F-actin

**Fig. 5.**
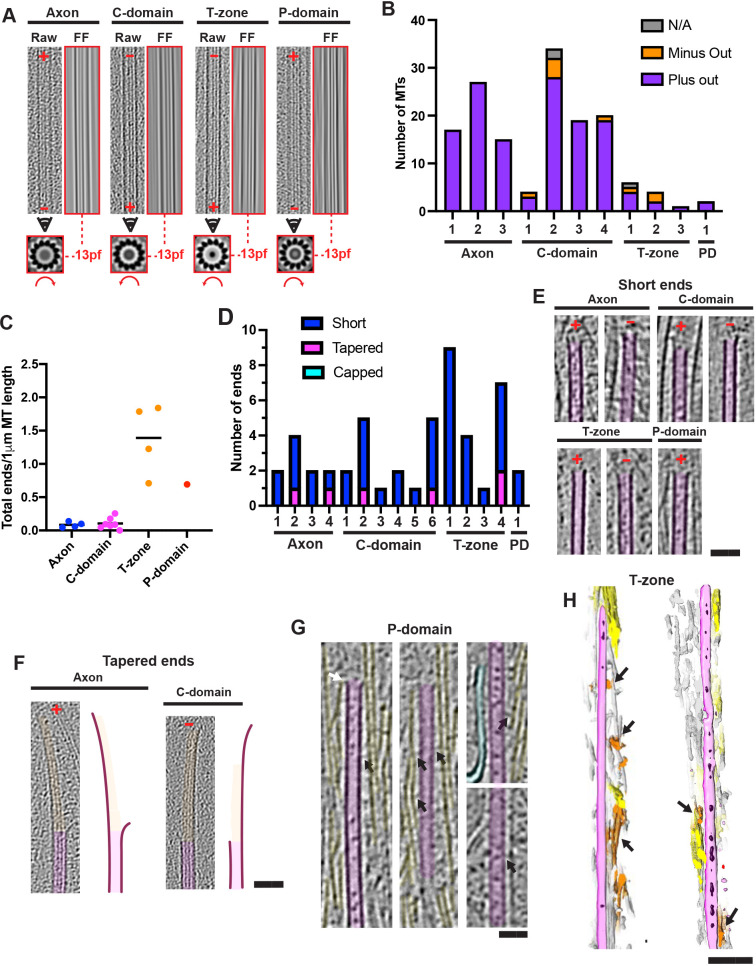
**Organisation and architectures of microtubules within growth cones.** (A) Representative examples of MTs illustrating protofilament number and polarity. Each set of 3 images includes a ∼30 nm thick longitudinal section through MT volume (2× binned, left) with corresponding image Fourier filtered at the origin (right, red box) showing 13-protofilament moiré patterns. Below, corresponding MT rotational average of 30 nm thick section viewed towards the cell periphery, showing protofilament number and handedness (curved red arrow). When viewed from the minus end or plus end, rotational average images exhibit clockwise or anticlockwise slew, respectively. In longitudinal sections, growth cone periphery is towards top; plus and minus end directions are indicated (red ‘+’ and ‘–’); consensus protofilament (pf) architecture is indicated between dashed red lines. (B) MT polarity relative to neuron periphery in individual tomograms. MTs assigned ‘N/A’ were perpendicular to the axon axis or bent ∼180°. Axon, 59 total plus-end peripheral MTs, 0 minus-end peripheral from 3 tomograms (each a different cell); C-domain, 69 total plus-end peripheral MTs, 6 minus-end peripheral, 2 N/A from 4 tomograms (each a different cell); T-zone, 7 total plus-end peripheral MTs, 3 minus-end peripheral, 1 N/A from 3 tomograms (each a different cell); P-domain (PD), 2 total plus-end peripheral MTs, 0 minus-end peripheral, 0 N/A from 1 tomogram. (C) MT ends frequency per 1 µm MT length in individual tomograms. Each data point represents a separate tomogram; axon (*n*=4), C-domain (*n*=7), T-zone (*n*=4), P-domain (*n*=1). Line indicates mean from all tomograms for each region. 50 MT ends were found in a total of 318 μm MT length. (D) Number of short and tapered ends in individual tomograms (number of tomograms of axon, *n*=4; C-domain, *n*=7, 1 tomogram contained no ends; T-zone *n*=4; P-domain, *n*=1). (E) Longitudinal views of short MT ends. (F) Longitudinal views of tapered MT ends. Short ends – 10 nm thick slices through 4× binned tomograms; tapered ends – 30 nm thick slices through 2× binned tomograms, false coloured in magenta and orange, respectively. Right: traced representations. Plus and minus ends are indicated (red ‘+’ and ‘–’). (G) Four longitudinal sections through regions of a single MT associated in parallel with P-domain F-actin bundles. Black and white arrows indicate crosslinks between MT shaft or tip and F-actin bundles, respectively; cyan indicates ER. (H) Two 3D sections (left and right) through T-zone segmentations; black arrows indicate transverse F-actin bundles running perpendicular on dorsal and ventral surfaces of periphery-orientated MTs. Segmentation colouring is as in Fig. 4A. Scale bars: E–G, 50 nm; H, 100 nm.

MTs were most prevalent in the C-domain (Fig. S3A–C), and all observed MTs throughout the growth cone exhibited a canonical 13-protofilament architecture (*n*=141, 11 tomograms) (Fig. 5A). Using the handedness of MT transverse segment rotational averages (Bouchet-Marquis et al., 2007; Sosa and Chretien, 1998), we could also show that the vast majority of growth cone MTs were orientated with their plus ends directed towards the periphery with only a few exceptions (Fig. 5B), as found previously in murine axons (Baas and Lin, 2011). A further subset of MTs in the T-zone and C-domain were either short, straight and perpendicular to the axonal axis (with both ends visible) or were long and curved backwards almost ∼180°, and thus neither of their ends pointed peripherally [Fig. 5B (N/A subset); Fig. S3A].

The highest frequency of MT ends as a proportion of MT length was in the T-zone (Fig. 5C). The ends were mainly limited in extent rather than extended, with protofilaments that were either blunt, somewhat flared, or gently curved. MT ends with long tapers (>50 nm) of mainly gently curved sheets were occasionally observed, which also sometimes contained flared protofilaments (Gudimchuk and McIntosh, 2021) (Fig. 5D–F). Of the six gently curved MT end tomograms, three could be determined to be plus ends (two from the axon, one from the C-domain) and one from a minus end (C-domain) (Fig. 5D,F). No examples of capped MTs were observed, consistent with the absence of γ-tubulin ring complexes (γ-TuRCs) in growth cones (Stiess et al., 2010).

In both the T-zone and the P-domain, densities were observed linking the sides of MTs to parallel large axial F-actin bundles, both along their lattice and at their ends (Fig. 5G). In addition, smaller F-actin bundles orientated roughly perpendicular to these MTs were often found in close proximity to their dorsal and ventral surfaces (Fig. 5H). Our cryo-ET data thereby provide evidence of a diversity of interactions that could mediate integration between the MT and actin filament systems.

### Lumenal particles and lattice defects in growth cone microtubules

**Fig. 6.**
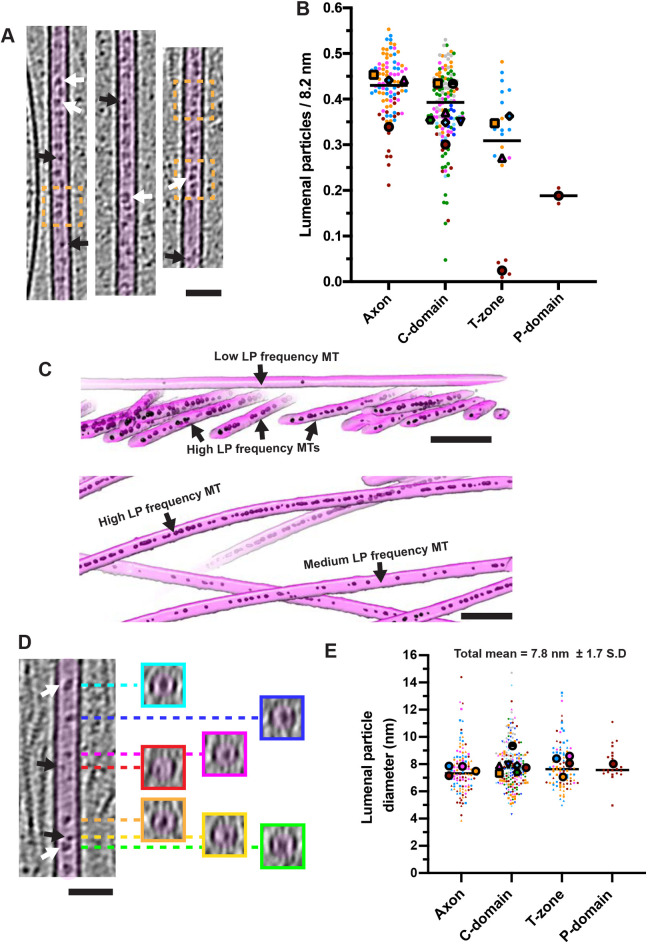
**Lumenal particles in neuronal growth cone MTs.** (A) Longitudinal views (2× binned) of MT lumenal particles. Orange dashed boxes indicate regions where particles are every ∼8 nm. White arrows, larger ring-like particles ∼7–10 nm diameter, black arrows, smaller particles ∼3–5 nm in diameter. (B) Super-plot of lumenal particle frequency per 8.2 nm. Each small data point represents a separate MT coloured by tomogram, mean frequency for individual tomograms are indicated with large coloured shapes, and lines indicate the overall medians. Overall means: Axon, 0.43±0.07 nm, *n*=94 MTs from 4 tomograms; C-domain, 0.38±0.09 nm, *n*=134 MTs from 7 tomograms; T-zone, 0.26±0.16 nm, *n*=22 MTs from 4 tomograms; P-domain, 0.19±0.02 nm, *n*=2 MTs from 1 tomogram. Values represent the mean±s.d. (C) Representative 3D segmentations of C-domain MTs (magenta) and lumenal particles (LP, black) illustrating particle frequency variability. (D) MT as in panel A, with transverse sections (∼5 nm depth) at positions indicated by dashed coloured lines. White arrows, larger ring-like particles ∼7–10 nm diameter; black arrows, smaller particles ∼3–5 nm diameter. (E) Super-plot of lumenal particle diameters. Each small data point represents a separate lumenal particle coloured by tomogram. Different small data point shapes indicate different MTs within each tomogram. Large coloured shapes indicate mean particle diameters within individual tomograms, lines indicate overall medians. Overall means: Axon, 7.6±1.7 nm, *n*=122 from 4 tomograms; C-domain, 7.9±1.7 nm, *n*=217 from 7 tomograms; T-zone, 7.9±1.6 nm, *n*=97 from 4 tomograms; P-domain, 8.0±1.3 nm, *n*=21 from 1 tomogram. Values represent the mean±s.d. Scale bars: A,D, 50 nm; C, 100 nm.

The MTs in our tomograms frequently exhibited particles within their lumens, as has been previously described (Atherton et al., 2018; Foster et al., 2022; Garvalov et al., 2006). We refer to these here as ‘lumenal particles’, rather than MT inner proteins (MIPs), since in many cases we cannot visualise a direct association with the MT inner wall, a defining characteristic of MIPs (Nicastro et al., 2006) (Fig. 6A). These lumenal particles are not uniformly distributed in the growth cone (Fig. 6B); MTs in the axon and C-domain – where MTs are denser – generally exhibited more lumenal particles (Fig. 6B), whereas they became increasingly sparse in the T-zone and P-domain. However, in some growth cone regions – particularly T-zones – the particle density in different tomograms varied substantially (Fig. 6B). Furthermore, within the same tomogram, neighbouring MTs – and even parts of the same MT (Fig. 6A) – exhibited different particle densities (Fig. 6C), with the most densely packed regions containing a particle roughly every 8 nm. Particle frequency did not obviously differ between curved and straight MT regions or with proximity to MT ends (Fig. 6C, Fig. 5E; Fig. S3).

The lumenal particles themselves were not identical (Fig. 6A,D,E). Although the mean lumenal particle diameter was 7.8±1.7 nm (mean±s.d.), the particles ranged from 3.9 to 14.7 nm, including ring-shaped structures with a diameter of 7–10 nm (Fig. 6D, white arrows) also described by Foster et al. (2022), as well as smaller, more globular particles of 4–6 nm diameter (Fig. 6D, black arrows). The average size corresponded roughly to a molecular mass of ∼200 kDa (with a range of 15 to ∼1400 kDa; Erickson, 2009). When MTs were viewed in cross-section, particles were not specifically localised within the lumenal space – some were located centrally whereas others appeared to associate with the inner surface of the MT lattice (Fig. 6D).

**Fig. 7.**
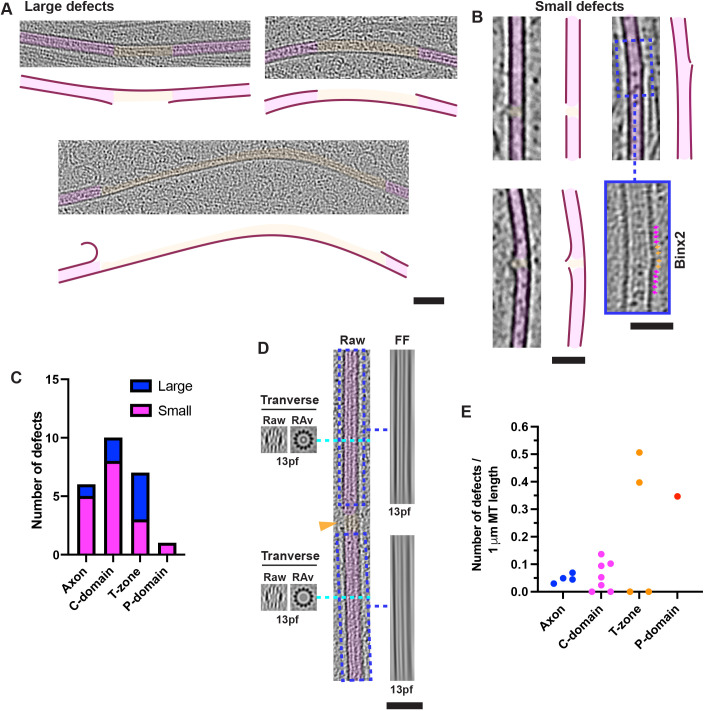
**MT lattice defects and discontinuities.** (A,B) Longitudinal views of large (>16 nm long; 30 nm slice, 2× binned) (A) and small (<16 nm long; 10 nm slice, 4× binned) (B) MT lattice defects in growth cones, false coloured in magenta and orange, respectively; traced representation shown alongside for clarity. Blue boxed insert in panel B, 2× binned (5 nm thick) version of the blue dashed boxed region above, with magenta and orange arrows pointing to tubulin monomers in lattice and a defect, respectively. (C) Absolute number of small (<16 nm) and large (>16 nm) defects (number of tomograms of axon, 4; C-domain, 7; T-zone, 4; P-domain, 1). (D) Centre, raw ∼30 nm thick longitudinal MT slice including an MT defect (orange arrowhead). Right: Fourier filtered (at origin, FF) images of blue dashed boxed regions in centre panel showing 13-protofilament (pf) moiré patterns either side of defect. Left: raw image and rotational average (RAv) of ∼10 nm thick MT transverse section, indicated with a cyan dashed line, indicating 13-protofilaments in both cases. (E) Frequency of defects per 1 µm MT length. Each data point represents a separate tomogram; axon, 4, C-domain, 7, T-zone, 4, P-domain, 1. Scale bars: A,B,D, 50 nm.

We also observed numerous defects and discontinuities in MT lattices in axons and growth cones (Fig. 7A,B). Some were very small, corresponding to one or two (16 nm) tubulin dimers, and apparently arising from subunits bending away from the MT axis (Fig. 7B). Others were larger (Fig. 7A), with the largest being ∼600 nm long. Larger defects were often associated with curved MT regions (Fig. 7A), and thus were more prevalent in the growth cone than the axon (Fig. 7C). No changes in protofilament number, MT architecture or polarity were observed on either side of the defects (Fig. 7D), as can occur *in vitro* (Atherton et al., 2018; Chretien and Fuller, 2000; Chretien et al., 1992; Schaedel et al., 2019). Relative to MT length, tomograms of T-zones exhibited the most variable MT defect frequency, with two T-zone tomograms exhibiting an MT defect at least every 2.5 µm of MT length (Fig. 7E). The axon and C-domain exhibited the narrowest range of defect frequency, with a defect being observed every 20 µm on average (Fig. 7E). There was no obvious relationship between the frequency and/or size of defects and the density of the adjacent lumenal particles (Fig. 7A,B).

### Cryo-ET of Dcx knockout neurons

**Fig. 8.**
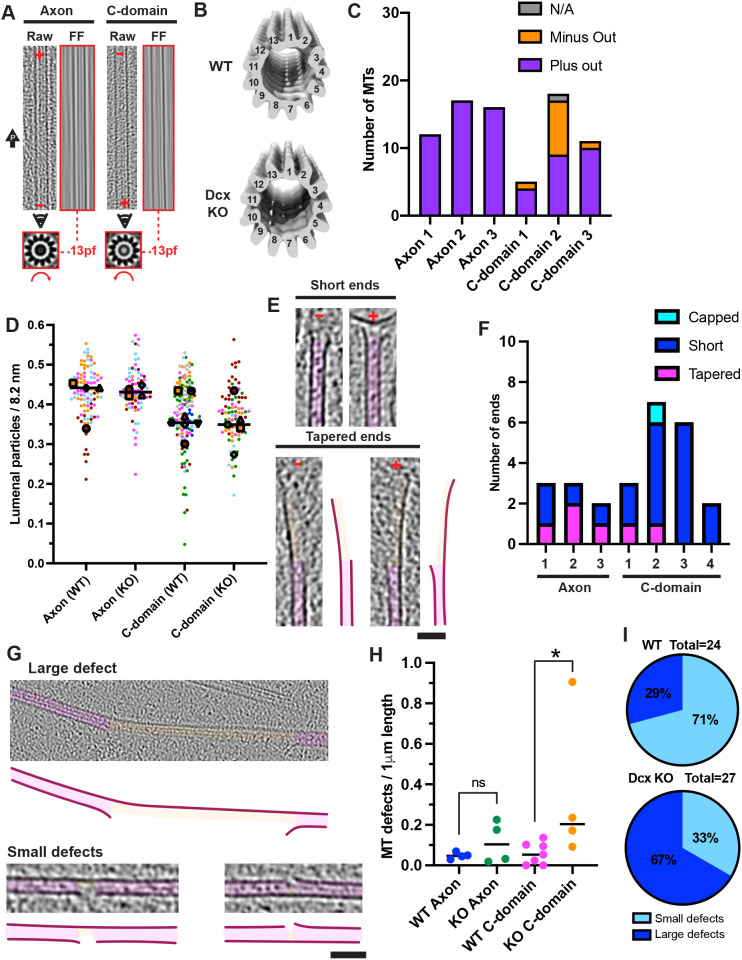
**Increased MT defects in doublecortin knockout neuronal growth cones.** (A) Representative images of Dcx KO neuron MTs showing protofilament number and polarity, as described in Fig. 7A. The arrow labelled ‘P’ indicates position of the cell front. (B) Transverse views (viewed from plus end) of sub-tomogram averages of random 13-protofilament MT subsets in WT (top, 1200 MT segments, 13 MTs) and Dcx KO (bottom, 1190 MT segments, 20 MTs) neurons. Volumes were low-pass filtered to estimated resolutions (Fig. S7C). (C) MT polarity relative to neuron periphery in individual tomograms. MTs assigned ‘N/A’ were either perpendicular to the axon axis or bent ∼180° such that both minus and plus directions were orientated peripherally. Axon, 45 plus-end peripheral MTs, 0 minus-end from 3 tomograms (3 axons); C-domain, 23 plus-end peripheral MTs, 10 minus-end, 1 N/A from 3 tomograms (3 neurons). (D) Super-plot of lumenal particle frequency per 8.2 nm. Each small data point represents a separate MT coloured by tomogram, mean frequency for individual tomograms indicated with large coloured shapes, lines indicate the overall medians. Overall means: WT axon, 0.43±0.07 nm, *n*=94 MTs, 4 tomograms; C-domain, 0.38±0.09 nm, *n*=134 MTs, 7 tomograms. Dcx KO axon, 0.43±0.06 nm, *n*=78 MTs, 4 tomograms; Dcx KO C-domain, 0.37±0.07 nm, *n*=84 MTs, 5 tomograms. Values represent the mean±s.d. (E) Longitudinal views (4× binned) of short and tapered MT plus and minus ends in Dcx KO growth cones. (F) Absolute numbers of short, tapered and capped MT ends in Dcx KO grow cones. Dataset size, total individual MT number in multiple tomograms (5 Dcx KO axon tomograms, 2 had no ends; 4 Dcx KO C-domain tomograms). (G) Longitudinal views (4× binned) of example MT defects in Dcx KO growth cones. (H) Frequency of defects per 1 µm MT length in WT and Dcx KO neurons. Each data point represents a separate tomogram; WT axon, 4; WT C-domain, 7; Dcx KO axon, 3; Dcx KO C-domain, 4. Mann–Whitney tests; WT C-domain versus Dcx KO C-domain, *P<0.05; WT C-domain versus Dcx KO C-domain with outlier data point removed, *P*=0.1083 (not significant); WT axon versus Dcx KO axon, *P*=0.89 (ns, not significant). (I) Numbers of large and small MT lattice defects as a percentage of total MT lattice defects in WT (top) and Dcx KO (bottom) pre-cone axons and C-domains. Dataset sizes indicate the total number of defects. Panels E and G: MTs and lattice defects are false coloured in magenta and orange, respectively; traced representation (right and bottom) shown for clarity. Scale bars: E,G, 50 nm.

DCX/Dcx is a modulator of neuronal growth cone morphology and the underlying MT and F-actin organisation (Bielas et al., 2007; Bott et al., 2020; Fu et al., 2013; Jean et al., 2012), and nucleates and stabilises 13-protofilament MTs *in vitro* (Moores et al., 2004). We performed cryo-ET of hippocampal neuron growth cones from Dcx knockout (KO) mice and analysed their cytoskeletal ultrastructure (Fig. 8; Figs S6 and S7). Like wild-type (WT) neurons, Dcx KO neurons exhibited classical morphology after culture for 2–3 days *in vitro* (Fig. S6A). By using cryo-ET, the general organisation of the pre-cone axon and growth cone in the Dcx KO neurons were indistinguishable from those in WT neurons (Fig. S6B–D). F-actin ultrastructure and organisation resembled that in WT cells (Fig. S7A,B) and filopodial F-actin bundles appeared indistinguishable from those in WT cells (Fig. S7B).

MTs in Dcx KO growth cones exhibited exclusively 13-protofilaments, as in WT neurons (Fig. 5A, Fig. 8A, Dcx KO, *n*=79, 7 tomograms), and sub-tomogram averages of both WT and Dcx MT segments were also consistent with a 13-protofilament architecture (Fig. 8B). In the axons of Dcx KO neurons, MTs were exclusively orientated with their plus ends towards the growth cone as seen in WT neurons (Fig. 8C, Fig. 5B). However, a higher overall proportion of Dcx KO C-domain MTs were orientated with their minus ends towards the neuronal periphery than WT C-domain MTs (29% versus 10%), although this distribution was variable between tomograms (Fig. 8C). Lumenal particle frequency was similar in axons and the C-domain of WT and Dcx KO neurons (Fig. 8D).

MT ends were almost exclusively uncapped in Dcx KO neurons (Fig. 8E,F; with a possible single exception, Fig. S7D). These MT ends are mainly short in appearance, although there appears to be a moderate increase in the proportion of tapered MT ends in the Dcx KO neurons (Fig. 8E,F versus Fig. 5D). A higher frequency of MT defects was observed in Dcx KO C-domains (Mann–Whitney test, *P*<0.05) compared with WT C-domains (Fig. 8G,H), although if the outlier data point is excluded from the Dcx KO data, this difference is not significant (Mann–Whitney test, *P*=0.1083), and we cannot exclude that this point arises, for example, from effects during preparation. However, a higher proportion of defects found in Dcx KO growth cones were >16 nm (Fig. 8I).

## DISCUSSION

Cryo-ET, neural network-driven density segmentation and sub-tomogram averaging revealed the distinct specialisations of the cytoskeleton in different parts of neuronal growth cones, reflecting the complexity of their behaviour and regulation. Within the tightly bundled actin filaments that dominate the P-domain (Fig. 3), different types of short F-actin crosslinkers – which our data cannot discriminate – likely coexist. However, filament dimensions, spacing and overall bundle architecture are most consistent with the involvement of fascin (Aramaki et al., 2016; Ishikawa et al., 2003; Jansen et al., 2011; Kureishy et al., 2002; Winkelman et al., 2016; Yang et al., 2013), and with fascin's known localisation and role in filopodia formation (Cohan et al., 2001; Sasaki et al., 1996). *In vitro*, fascin-mediated actin bundles exhibit a very similar overall architecture to those we observed, and are distinct when compared, for example, with α-actinin (Hampton et al., 2007) and espin-mediated bundles (Claessens et al., 2008). Intriguingly, fascin actin bundles *in vitro* exhibit a maximum size of ∼20 filaments (Claessens et al., 2008), which is limited by bundle packing. However, we and others (Aramaki et al., 2016) observed examples of much bigger bundles (>100 filaments) within filopodia. We also observed flexible actin filaments weaving between bundles, which, together with other actin modulators, may loosen bundle packing sufficiently to allow larger overall bundles. Larger but less stiff bundles may be important for the balance between the dynamic activities of filopodia and shape maintenance.

Alongside the filopodia-derived bundles in the P-domain and T-zone, we also observed shorter repeat length actin filaments. Although the resolution of our sub-tomogram reconstructions are limited, the ∼27 nm half helical repeat length, wider appearance and 3D reconstruction of these filaments suggest that cofilin is bound, consistent with its localisation to the P-domain and roles in filopodial F-actin severing and recycling (Bamburg and Bray, 1987; Breitsprecher et al., 2011; Gungabissoon and Bamburg, 2003; Marsick et al., 2010). *In vitro*, cofilin binds cooperatively to F-actin, alters the actin filament structure to a half helical repeat length of ∼27 nm and promotes ADP–actin filament severing (Bobkov et al., 2004; Galkin et al., 2001; Huehn et al., 2020; McCullough et al., 2008; McGough et al., 1997; Tanaka et al., 2018). When associated with bundles in our cryo-ET data, these short-repeat cofilin-like filaments were frequently peripheral, exhibited more positional fluidity within the bundle and often detached from the main filament cluster. They were also not regularly crosslinked to long-repeat filaments, likely due to mismatch between helical repeats. Our data provide good evidence that well-decorated cofilin-bound actin filaments exist in the P-domain and suggest that cofilin decoration may enable filament detachment from bundles before severing, thereby contributing to overall retrograde flow from the front of the cell (Vitriol et al., 2013).

Despite the cofilin-type activity in the P-domain, filopodia-derived bundles ultimately taper and terminate in the T-zone, often with bundles splitting apart and bending away into actin networks that run roughly perpendicular to filopodia bundles. How filaments are bent away from F-actin bundles is unclear; however, myosin-II is known to unbundle F-actin, contribute to severing, and produce retrograde flow and bending forces in the T-zone (Burnette et al., 2008; Ishikawa et al., 2003; Medeiros et al., 2006; Schaefer et al., 2002). Further, since filament peeling is observed in both directions from filopodia-derived bundles, the resulting anti-parallel networks necessary for T-zone myosin-II-mediated contractility can be formed. Consistent with this, myosin-II–F-actin *in vitro* complexes share striking similarities to the tapered unbundling observed here *in situ* (Haviv et al., 2008).

The density of T-zone F-actin networks suggests that they do not simply create a physical barrier to MTs entering the P-domain. However, close association of MTs with F-actin is consistent with the previously observed restriction of MT entry into the growth cone periphery primarily by retrograde flow (Burnette et al., 2008; Burnette et al., 2007; Kahn and Baas, 2016; Schaefer et al., 2002; Schaefer et al., 2008). Retrograde flow has also been shown to bend and break growth cone MTs, thereby contributing to their overall dynamics (Waterman-Storer and Salmon, 1997). However, a number of end-binding proteins, microtubule-associated proteins (MAPs) and motors important for neuronal migration form links to F-actin in growth cones (Cammarata et al., 2016; Geraldo and Gordon-Weeks, 2009; Kahn and Baas, 2016), and there is abundant evidence in our data of such MT–F-actin crosslinkers, which likely mediate cytoskeleton cross-talk (Fig. 5G,H).

Growth cone MTs contain diverse and unevenly distributed lumenal particles. As MTs in the periphery are more dynamic compared to the axon and C-domains, MTs with high and low lumenal particle densities may represent populations of stable and dynamic MTs, respectively; the more even distribution of lumenal particles in axon MTs is consistent with this idea (Foster et al., 2022). The identities of these lumenal particles remain unclear – some cytoplasmic components may simply be trapped during polymerisation. However, other densities are likely to be functionally relevant – for example, neuronal MAP6 is likely to be present (Cuveillier et al., 2020). Based on the volume of the MT lumen, the average particle volume, and their average frequency, we estimate that lumenal particles occupy roughly 7% of the total inner volume of neuronal MTs. However, their precise roles are unclear, and the MT lumenal space remains an intriguing and poorly understood cellular compartment.

At least a subset of growth cone MTs are known to be dynamic (Baas and Black, 1990; Edson et al., 1993; Tanaka et al., 1995). Strikingly, however, the large majority of MT ends we observed were relatively short, a few exhibited longer and gently curved sheet-like tapers, and we found no examples of extensively curled protofilaments that have been proposed to characterise both growing and shrinking MTs (McIntosh et al., 2018). The large number of neuronal MAPs may constrain the morphologies of dynamic MT ends such that curling protofilaments are less likely to form. MT lattice defects, and the repair processes they promote, also contribute to the regulation of MT dynamics (Thery and Blanchoin, 2021). In our data, MTs throughout the growth cone exhibit discontinuities or substantial breaks, particularly in the T-zone. Lumenal particle-forming MAP6 is known to induce MT defects *in vitro* (Cuveillier et al., 2020); however, in our data there was no clear correlation between defects and lumenal particle density, and defects were more common in peripheral regions with overall fewer particles. Despite the presence of these defects, the consistent cylindrical 13-protofilament architecture of growth cone MTs indicates that this property is tightly controlled, and the pleiotropic brain tubulin assemblies sometimes seen *in vitro* (for example, Guyomar et al., 2021 preprint) are not observed in our neurons. Large defects were frequently observed on curved MTs, and it seems likely that at least some larger defects arise from strain-induced MT fraying, whereas short MTs may represent remnants of broken MTs caused by retrograde flow (Burnette et al., 2008; Schaefer et al., 2002, 2008; Waterman-Storer and Salmon, 1997).

DCX/Dcx has a number of reported roles in migrating neurons related to its cytoskeleton regulation functions (Bielas et al., 2007; Deuel et al., 2006; Friocourt et al., 2003; Fu et al., 2013; Jean et al., 2012; Li et al., 2021; Tint et al., 2009). In Dcx KO neurons, growth cone architecture and the overall organisation and distribution of the cytoskeleton were comparable to those in WT neurons; this included F-actin organisation, in contrast to an earlier report (Fu et al., 2013). *In vitro*, whereas MTs polymerised from diverse tubulin sources including mouse brain tubulin (Vemu et al., 2017) polymerise with a range of protofilament architectures (Ray et al., 1993), DCX nucleates and selectively stabilises 13-protofilament MTs (Moores et al., 2004). In the Dcx KO growth cones, however, all MTs exhibited a 13-protofilament architecture as in the WT neurons, highlighting the role of diverse cellular factors in addition to Dcx in ensuring uniformity of MT architecture even in the absence of γ-TuRCs (Stiess et al., 2010). However, in our Dcx KO tomograms, C-domain MTs exhibited a higher proportion of MTs orientated with their minus ends towards the neuronal periphery, and a higher proportion of large compared to small defects. DCX/Dcx is highly expressed in growth cones in which it has been shown to stabilise and straighten MTs (Jean et al., 2012; Tint et al., 2009), and its binding is modulated by MT curvature (Bechstedt et al., 2014; Ettinger et al., 2016). Taken together with our data, this suggests that the absence of Dcx may lead to a higher frequency of curvature-induced MT fraying or breakage in C-domains, where Dcx is particularly abundant (Fig. S1B).

How might this potential role for DCX/Dcx explain brain developmental phenotypes caused by its absence? Immature neurons experience substantial compression forces during navigation through the layers of the developing brain (Tyler, 2012); the requirement to maintain and repair ruptured MTs during migration may be particularly acute, and DCX/Dcx may be involved in this process. Cryo-ET will continue to be a vital tool for the molecular visualisation and dissection of machinery involved in key cellular processes including responses to guidance signals and injury in neurons. Insights arising from such studies will also inform developments of new treatments for neuronal damage and degeneration (Griffin and Bradke, 2020).

## MATERIALS AND METHODS

### Transgenic mice and genotyping

WT and Dcx KO mice were maintained on the Sv129Pas background with more than ten generations of backcrosses (Kappeler et al., 2006, 2007). Genotyping was performed by PCR to verify the inactivation of the Dcx gene in KO animals (Kappeler et al., 2006). All experiments were performed in accordance with institutional, national and international guidelines (EC directive 2010/63/UE) and were approved by the local ethical committees (French MESR N°: 00984_02). The day of confirmation of vaginal plug was defined as embryonic day (E)0.5.

### Neuronal cell dissociation and isolation

Embryonic mouse hippocampus was dissected at E17.5 in ice-cold 0.02 M HEPES in Ca^2+^/Mg^2+^-free Hanks’ Balanced Salt Solution (HBSS, Gibco) and primary cell suspensions dissociated via incubation with 2.5 mg/ml trypsin (Sigma). After centrifugation to a pellet (5 min, 300 ***g***), cells were resuspended in culture media for light or electron microscopy experiments as indicated below.

### Cell culture for fluorescence microscopy

For fluorescence microscopy experiments, dissociated neuronal pellets were resuspended and cultured in Neurobasal medium supplemented with B27 (1%, Gibco), GlutaMAX (2 mM, Gibco), penicillin (100 units/ml, Invitrogen) and streptomycin (100 mg/ml, Invitrogen). Only mouse litters comprising at least one WT and one Dcx KO animal were used. Dissociated cells were plated on 14 mm-diameter coverslips (0.3×10^5^ cells/coverslip) coated with poly-L-lysine (0.05 mg/ml, Sigma) and laminin (0.01 mg/ml, Invitrogen) and cultured at 37°C in the presence of 5% CO_2_.

### Fluorescence microscopy

Neurons were fixed after 3 days *in vitro* (DIV) following the protocol from (Tint et al., 2009), applying PEM buffer (100 mM PIPES, 5 mM EGTA, 2 mM MgCl_2_, pH 6.8) with 0.3% glutaraldehyde, 0.1% IgepalCA630 (Nonidet P-40, Sigma-Aldrich, N-3516) and 10 µM taxol (Sigma-Aldrich, T1912) for 10 min at room temperature (RT). Coverslips were then washed five times with PBS, once for 15 min in PBS with 0.5% Triton X-100, twice for 7 min each with 10 mg/ml sodium borohydride in PBS, and finally five more times with PBS. Neurons on coverslips were incubated with blocking solution (5% normal goat serum, 0.1% Triton X-100 in 1× PBS) for 1 h at RT, followed by incubation with primary antibodies for Dcx (1:1000, Francis et al., 1999) and neuron-specific β3-tubulin (1:1000, TUJ1, RD Biosystems) in blocking solution for 2 h at RT or overnight at 4°C. Secondary antibodies [goat anti-mouse Alexa Fluor 555 (1:1000, A32727, Thermo Fisher), goat anti-rabbit Alexa Fluor 488 (1:1000, A-11008, Thermo Fisher)] were incubated in blocking solution combined with Hoechst for 60 min at RT in the dark, followed by a 60 min incubation with phalloidin–Alexa Fluor 633 (1:100, A22284, Life Technologies) in PBS. Coverslips were mounted with Fluoromount G (Southern Biotechnology).

### Neuronal cell culture and preparation for cryo-EM

For electron microscopy experiments, dissociated neuronal pellets were resuspended in Hibernate-E medium (Gibco) supplemented with B27 (Invitrogen) and Pen-Strep (100 units/ml penicillin and 100 mg/ml streptomycin, Invitrogen) and shipped overnight from Paris to London in the dark at 4°C in Hibernate-E media. Quantifoil R2/4 gold G200F1 Finder EM grids were placed on the surface of plastic culture dishes and sterilised under UV light. The dishes and overlaid EM grids were then coated with poly-D-lysine (Millipore) and mouse laminin (Thermofisher). Within 24–30 h of initial dissociation, the neurons were pelleted by centrifugation (5 min, 300 ***g***), then resuspended and plated in Neurobasal medium with B27 and Pen-Strep and cultured at 37°C in the presence of 5% CO_2_ for 2–3 days *in vitro* without media change until the characteristic morphology was observed using phase-contrast light microscopy. Grids were extracted from culture dishes, 3 µl of conditioned culture media added, blotted for 8–10 s and plunge vitrified in liquid ethane using a Vitrobot (FEI) set at 37°C and 80% humidity.

### Cryo-ET data collection

Cryo-ET was performed using a Tecnai G2 Polara operating at 300 kV with a Quantum post-column energy filter (Gatan), in zero-loss imaging mode with a 20 eV energy-selecting slit. Single-axis cryo-ET was performed at 3–6 µm defocus with a K2 Summit direct electron detector (Gatan) operating in counting mode (at 5 e^-^ per pixel per second) with a final sampling of 5.38 Å per pixel. Dose-symmetric tilt-series (Hagen et al., 2017) within a −60° to +60° tilt range were collected at 3° increments to give a total dose of 110 e^-^/Å^2^. Movies of three or four subframes per second were collected at each tilt and aligned on-the-fly using MotionCor2 (Zheng et al., 2017). 30 or 16 tilt series of a ∼2.15 by 2.15 μm field of view were collected from different WT (derived from 12 neurons, 7 different mice and 10 different grids) and Dcx KO (derived from 9 neurons, 4 different mice and 6 different grids) growth cone regions, that lay over 2 μm diameter holes in the carbon substrate of the EM grid (Fig. S1C).

### Cryo-ET image processing

Fiducial-less alignment via patch tracking was performed on each tilt series in the Etomo graphical user interface to IMOD v.4.9.0 (Kremer et al., 1996). The contrast transfer function (CTF) was determined at each tilt using CTFFIND4 (Rohou and Grigorieff, 2015), and then a dose-weighted aligned tilt-series was produced using Summovie (Grant and Grigorieff, 2015) under the control of custom scripts. Three-dimensional CTF correction and tomogram reconstruction was performed by weighted back projection of dose-weighted tilt series with novaCTF (Turonova et al., 2017) using custom scripts.

Semi-automated tomogram segmentation was performed using the tomoseg module of EMAN2 v.2.2 (Chen et al., 2017). 4× binned tomograms with low frequencies amplified via a positive B-factor of 100,000 were used for training and segmentation purposes. Example tomogram regions containing the target feature (positive references) or not containing the target feature (negative references, including regions containing similar features that could give rise to false positives) were selected, then annotated and used as input for training neural networks. Trained networks were applied to multiple tomograms and segmentations were then cleaned manually, both by using inverted masks derived from segmentations of other features to remove false positives, and by further manual cleaning using the ‘volume eraser’ and ‘hide dust’ tools in Chimera (Pettersen et al., 2004) (Fig. S2A,B). Segmentation accuracy and specificity was verified by experimenter visual comparison to raw tomograms (Fig. S2C).

Subtomogram averaging of MT and F-actin filaments was performed in Dynamo. 341 volumes of 68 by 68 by 68 nm were picked from 4× binned tomograms using filament model tools. Initial 40 Å low-pass filtered references were generated from data aligned only along filament axes. Initial coarse alignment was performed using 4× binned data, before finer alignment with 1× binned data.

## Supplementary Material

Supplementary information

Reviewer comments
