# Supplementary material for: Visualising the cytoskeletal machinery in neuronal growth cones using cryo-electron tomography
Source: J Cell Sci. 2022 Apr 8;135(7):jcs259234. doi: 10.1242/jcs.259234 (PMC9016625; doi:10.1242/jcs.259234)
Supplement: Supplementary information [file joces-135-259234-s1.pdf]

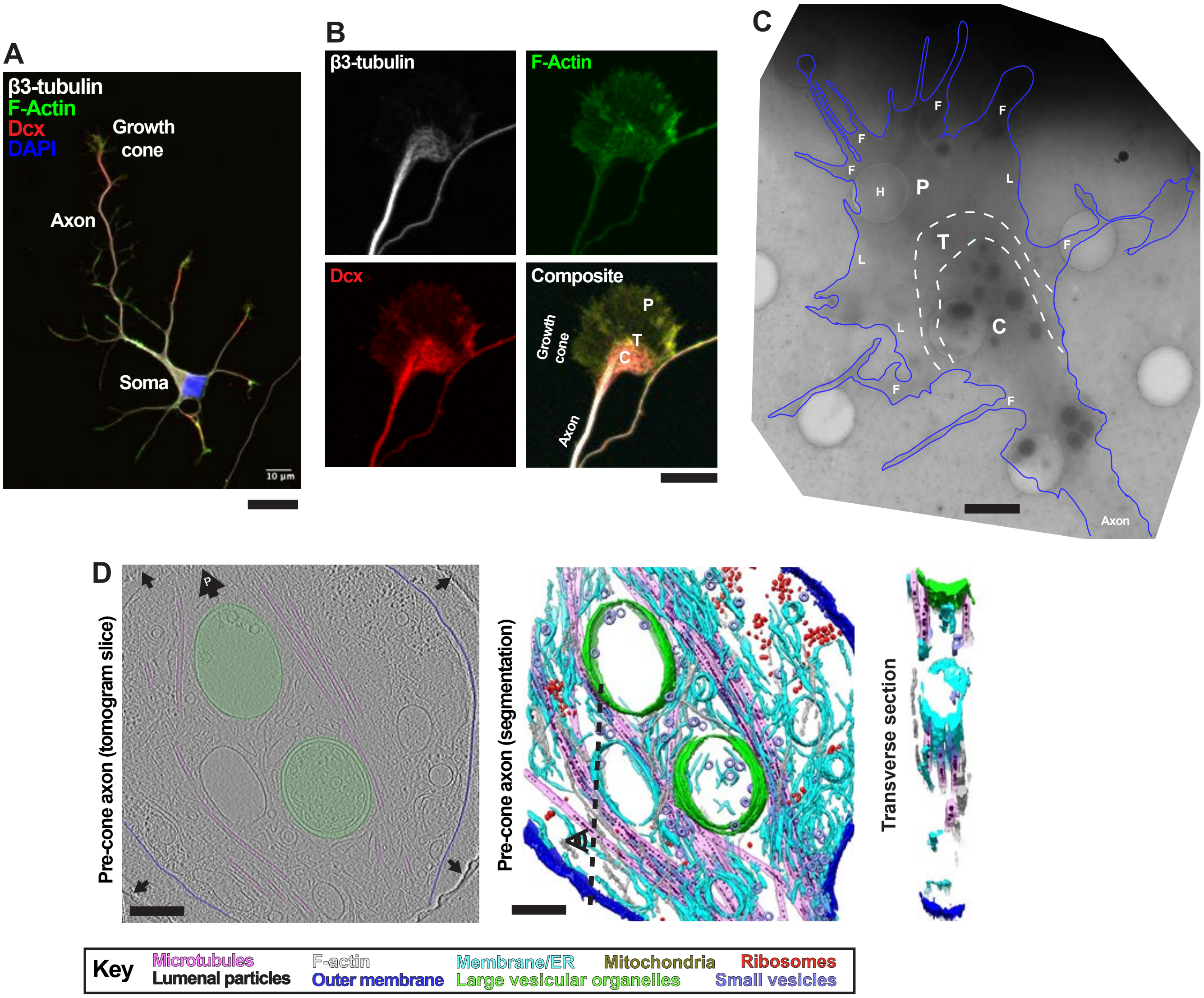

Journal of Cell Science • Supplementary information

Fig. S1. Cryo-electron tomography of primary neuronal growth cones and axons.

(A) Mouse primary hippocampal neuron cultured for 3 days in vitro, demonstrating the characteristic polarised stage 3 morphology, labelled as indicated with DAPI (nucleus) and phalloidin (F-actin) dyes and antibodies to β3-tubulin and Dcx; **(B)** Fluorescence light microscopy images of a mouse hippocampal neuron growth cone labelled as indicated with phalloidin (F-actin) dye and antibodies to β3-tubulin and Dcx; the central domain **(C)**, transition zone (T) and peripheral domain (P) are indicated on the composite image. (C) Low magnification cryo-EM image of a growth cone. The cell membrane is depicted in blue; the C-domain (C), T-zone (T) and Pdomain (P) are indicated; F, filopodia; L, Lamellipodia; H, underlying holes in the carbon substrate; I, Ice contamination. **(D)** Example tomogram slice and corresponding segmentation from an axonal region. Left panel shows central section through a binned x 4 tomogram, with MTs (magenta), large vesicular organelles (green) and outer membrane (blue) indicated with overlaid false colouring. Direction of the leading edge is indicated with a large black arrow containing a 'P' and edges of the carbon substrate hole are indicated with small black arrows. The middle panel shows the corresponding whole volume 3D semi-automated segmentations. The right panel shows a transverse section of the segmented region indicated with a dashed line in the corresponding central panel (section viewing direction also indicated in central panel). Segmentation false colouring is according to the key. Scale bars: (A) = 20 µm, (B) = 10 µm, (C) = 2 µm, D = 200 nm.

Journal of Cell Science • Supplementary information

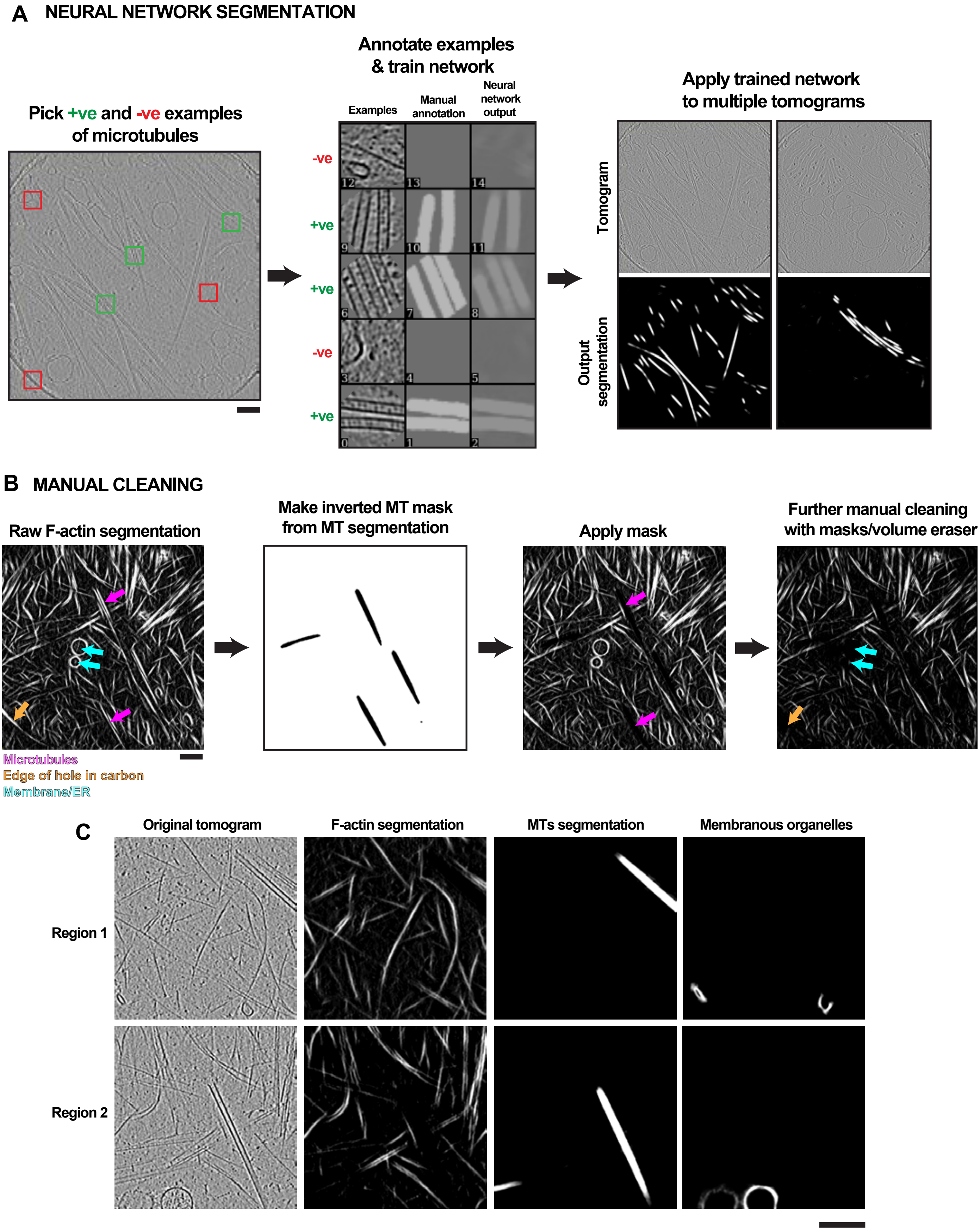
A
NEURAL NETWORK SEGMENTATION
Pick +ve and -ve examples of microtubules
Annotate examples & train network
Examples
Manual annotation
Neural network output
-ve
+ve
+ve
-ve
+ve
Apply trained network to multiple tomograms
Tomogram
Output segmentation
B
MANUAL CLEANING
Raw F-actin segmentation
Make inverted MT mask from MT segmentation
Apply mask
Further manual cleaning with masks/volume eraser
Microtubules
Edge of hole in carbon
Membrane/ER
C
Original tomogram
F-actin segmentation
MTs segmentation
Membranous organelles
Region 1
Region 2

Fig. S2. Semi-automated tomogram segmentation methodology.
(A) Steps performed during initial neural network training using the tomoseg module of EMAN2 v.2.2 (Chen et al., 2017), using segmentation of MTs as an example. First, true examples of the target feature (green boxes) and false examples including similar features (red boxes) are picked (left panel). Next, the true examples are annotated manually, and the neural network is trained (centre panel). This step is repeated until the neural network output faithfully recognises the target feature with minimal false positives. The trained neural network is then applied to recognise the feature in multiple tomograms. **(B)** Illustration of the manual cleaning steps performed following neural network segmentation. F-actin segmentation is used as an example, where the most successful neural network training process also produced many false positives (left panel, indicated by arrows coloured according to the key below) requiring manual removal; central two panels: MTs were removed by making an inverted MT segmentation mask and applying it to the F-actin segmentation (central two panels); right panel: finally, density for other false positives was removed manually using the volume eraser tool in Chimera (Pettersen et al., 2004). **(C)** Exemplar tomogram regions and corresponding final F-actin, MT and membranous organelle segmentations showing good segmentation accuracy and efficiency. Scale bars: A-C = 200 nm.

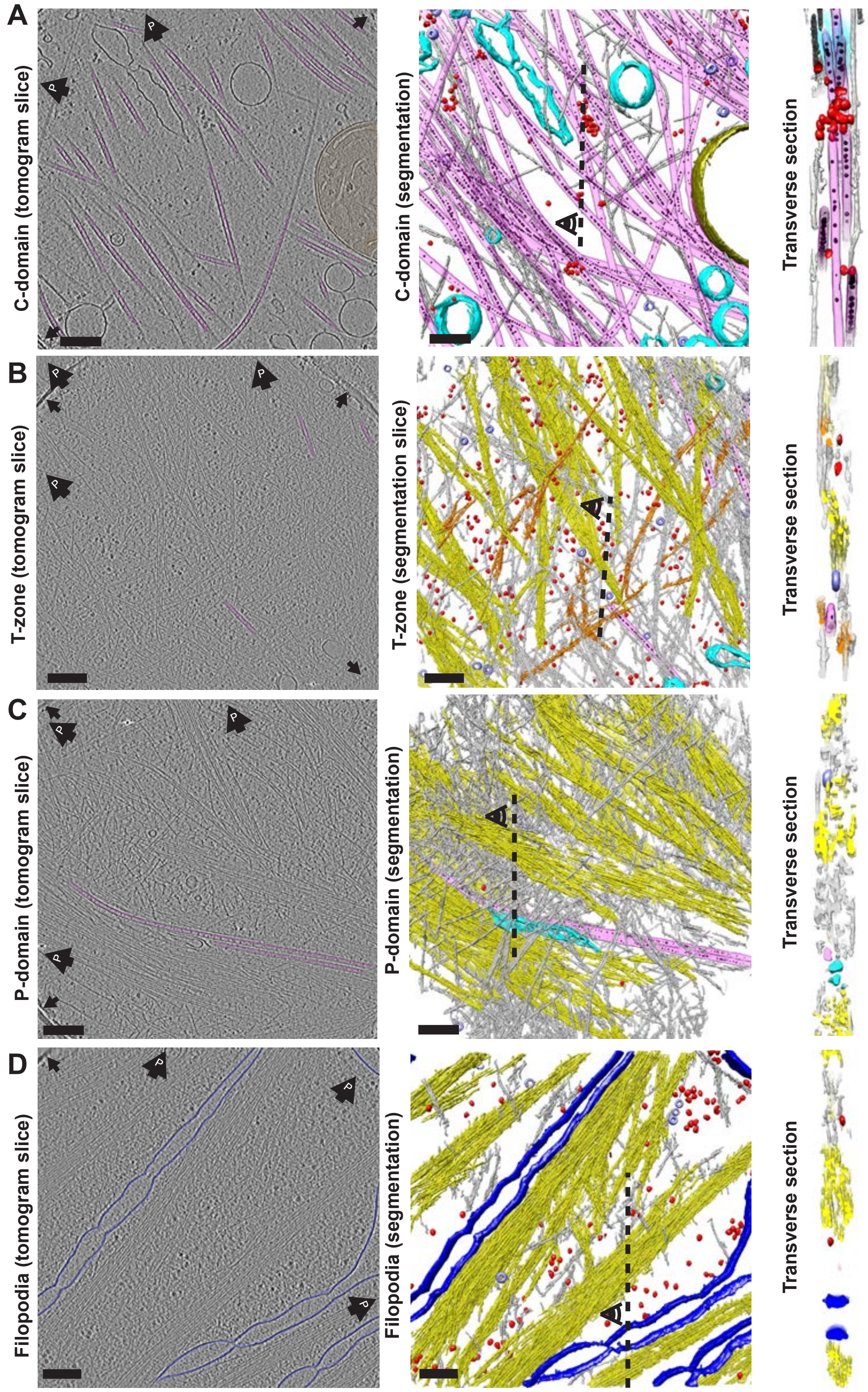

Journal of Cell Science • Supplementary information

Fig. S3. The molecular landscape of neuronal growth cones.
Example tomograms and corresponding segmentations from different growth cone regions; **(A)** C-domain, **(B)** T-zone, **(C)** P-domain, **(D)** P-domain filopodia. Left panels show central sections through binned x 4 tomograms, with MTs (magenta) and outer membrane (blue) indicated with overlaid false colouring. Direction of the leading-edge distal periphery is indicated with large black arrows containing a ‘P’ and edges of the carbon substrate hole are indicated with small black arrows. Middle panels show corresponding whole volume 3D semi-automated segmentations. Right panels show transverse sections of the segmented regions indicated with dashed lines in corresponding central panels (section viewing direction also indicated in central panels). Segmentation colouring is according to the key. Scale bars = 200 nm.

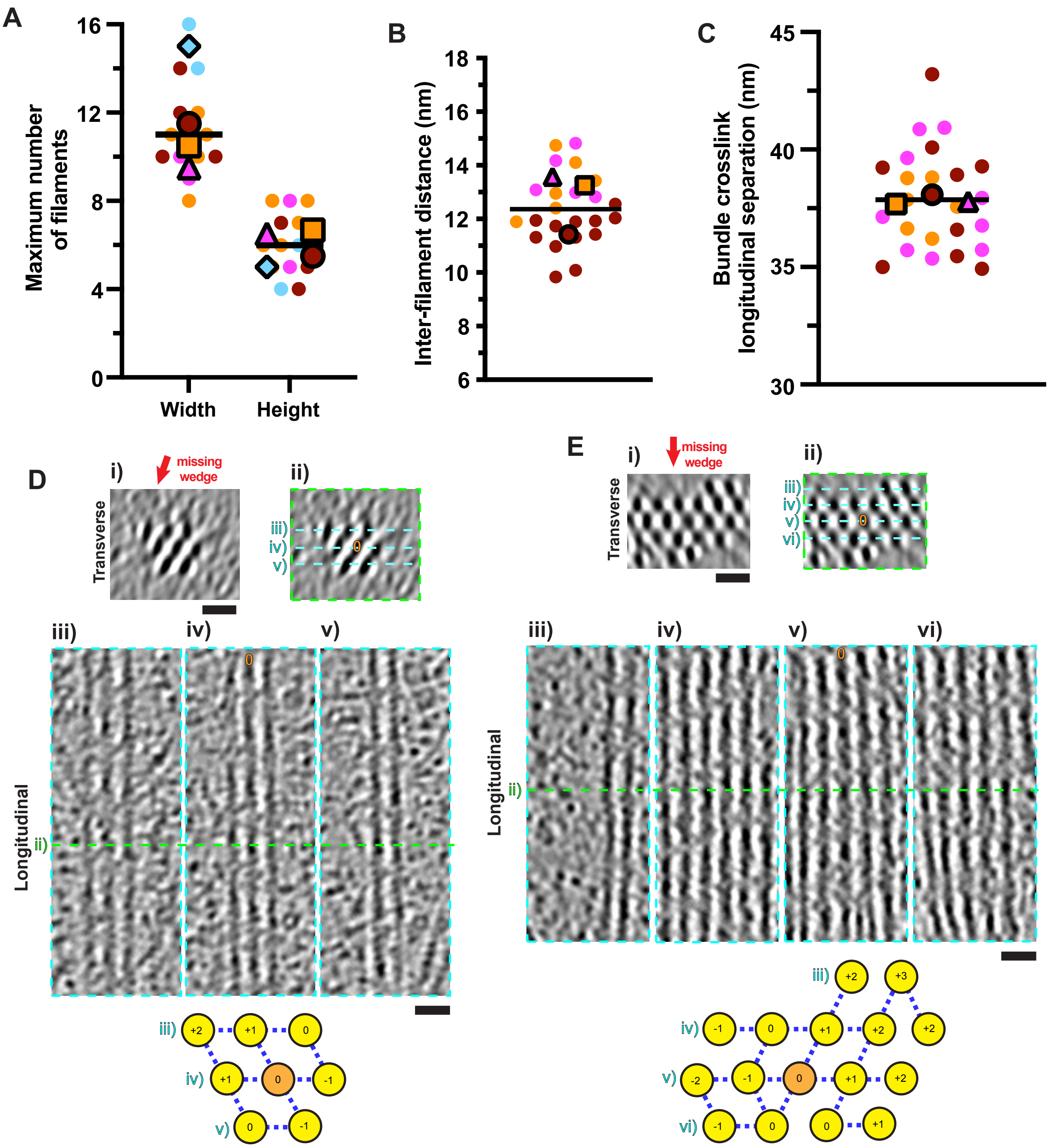
A
Maximum number of filaments
16
12
8
4
0
Width
Height
B
Inter-filament distance (nm)
18
16
14
12
10
8
6
C
Bundle crosslink longitudinal separation (nm)
45
40
35
30
D
i)
missing wedge
Transverse
ii)
iii)
iv)
v)
0
Longitudinal
E
vi)
+2
+3
+1
-1
-2

Fig. S4. Ultrastructure of the ordered P-domain F-actin arrays.
(A) Super-plot (Lord et al., 2020) of maximum filament number in width and height of P-domain bundles. Each data point represents an individual filopodial F-actin bundle (N = 14 from 4 tomograms, shown as different colours), with a line indicating the overall median. Mean values for each tomogram are shown in their respective colours with larger shapes. Overall mean width, 11.3 ± 2.2 S.D, mean height 6.1 ± 1.4 S.D filaments. **(B)** Super-plot of inter-filament distances in P-domain F-actin bundles. Each data point represents a separate adjacent filament pair (N = 23 from 3 tomograms, shown as different colours). Mean values for each tomogram are shown in their respective colours with larger shapes. Line indicates the overall mean (12.4 nm ± 1.3 S.D). **(C)** Super-plot of regular longitudinal crosslink separation distance in P-domain F-actin bundles. Each data point represents an individual pair of adjacent cross-links along the F-actin longitudinal axis (N = 25 from 3 tomograms, shown as different colours). Mean values for each tomogram are shown in their respective colours with larger shapes. Line indicates the overall mean (37.9 nm ± 2.1 S.D).
(D-E) Repeat shift analysis for two separate hexagonal F-actin bundle arrays, as in Fig. 3B.

Journal of Cell Science • Supplementary information

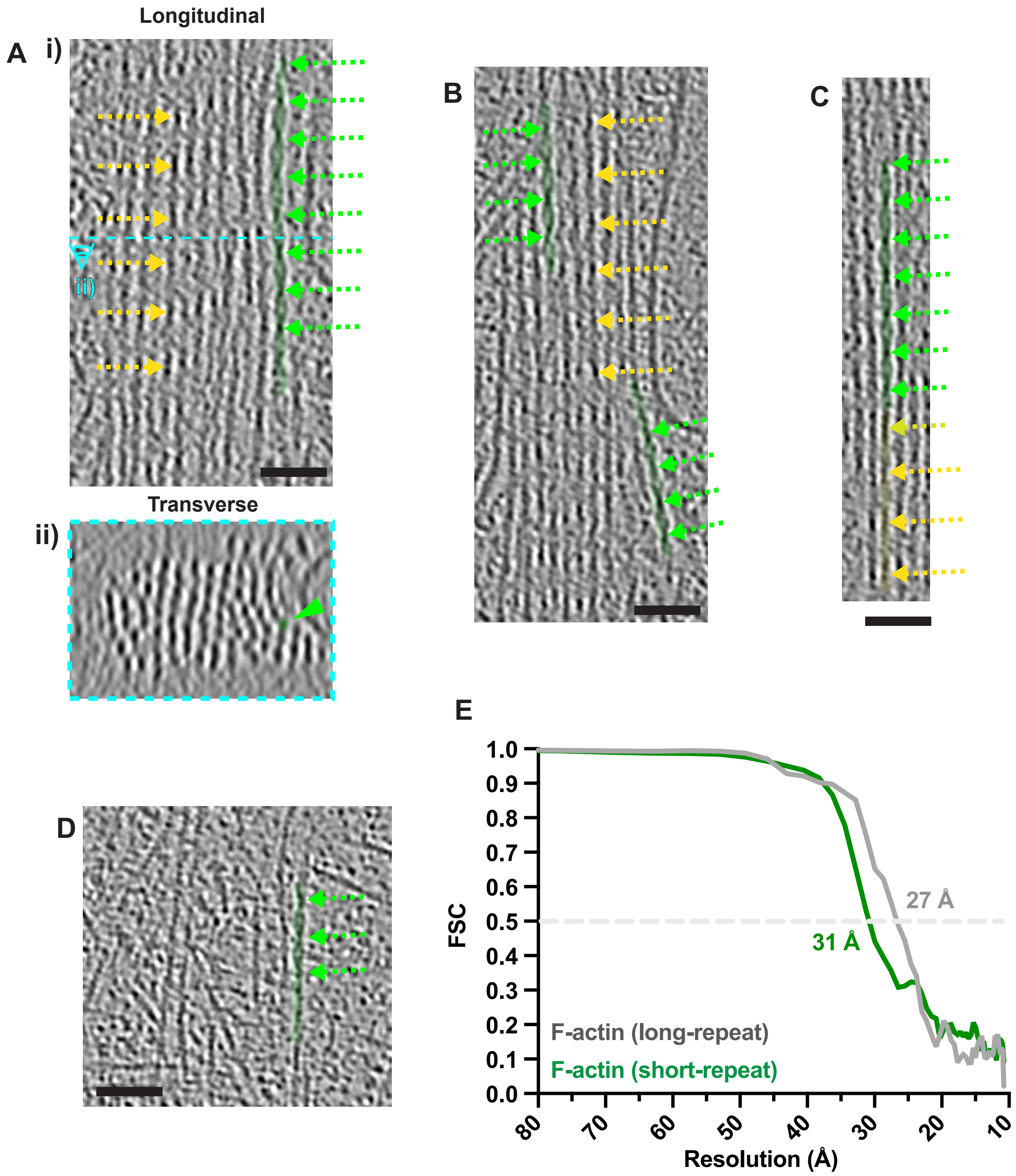

Longitudinal
A
i)
ii)
Transverse
ii)
B
C
D
E
FSC
1.0
0.9
0.8
0.7
0.6
0.5
0.4
0.3
0.2
0.1
0.0
27 Å
31 Å
F-actin (long-repeat)
F-actin (short-repeat)
80
70
60
50
40
30
20
10
Resolution (Å)

Fig. S5. Characteristics of short-repeat F-actin in the Pdomain and T-zone
(A) i) Longitudinal and ii) transverse (~20 nm depth) views of a large F-actin bundle in a 2x binned P-domain tomogram. Dashed cyan line in i) illustrates position of transverse section in ii). Examples of ~37 nm F-actin half helical repeat lengths are indicated with yellow dashed arrows, while shorter, rarer ~27 nm F-actin half helical repeat lengths are shown with green dashed arrows.
A short-repeat filament is false coloured in green and additionally indicated in ii) with a green arrowhead. In this and subsequent longitudinal views, more peripheral and more central regions of the neuron are at the top and bottom of the images respectively. **(B)** Longitudinal view of a large F-actin bundle in a 2x binned P-domain tomogram, with two short-repeat filaments false coloured in green. F-actin half helical repeat lengths are indicated with arrows as in panel A. **(C)** Longitudinal view of a large F-actin bundle in a 2x binned P-domain tomogram, showing a single filament transitioning from short to long-repeat (with short and long-repeat filament segments false coloured in green and yellow respectively). F-actin half helical repeat lengths are indicated with arrows as in panel A. **(D)** Longitudinal view of an individual non-bundled short-repeat F-actin filament in a 2x binned T-zone tomogram, false coloured in green. F-actin half helical repeat lengths are indicated with arrows as in panel A. (E) Fourier Shell Correlation (FSC) curves generated from half sets for subtomogram-averages of long-repeat Factin filaments (grey) and short-repeat F-actin filaments (green). Resolutions are indicated at the FSC = 0.5 criterion. Scale bars: A - D = 40 nm.

Journal of Cell Science • Supplementary information

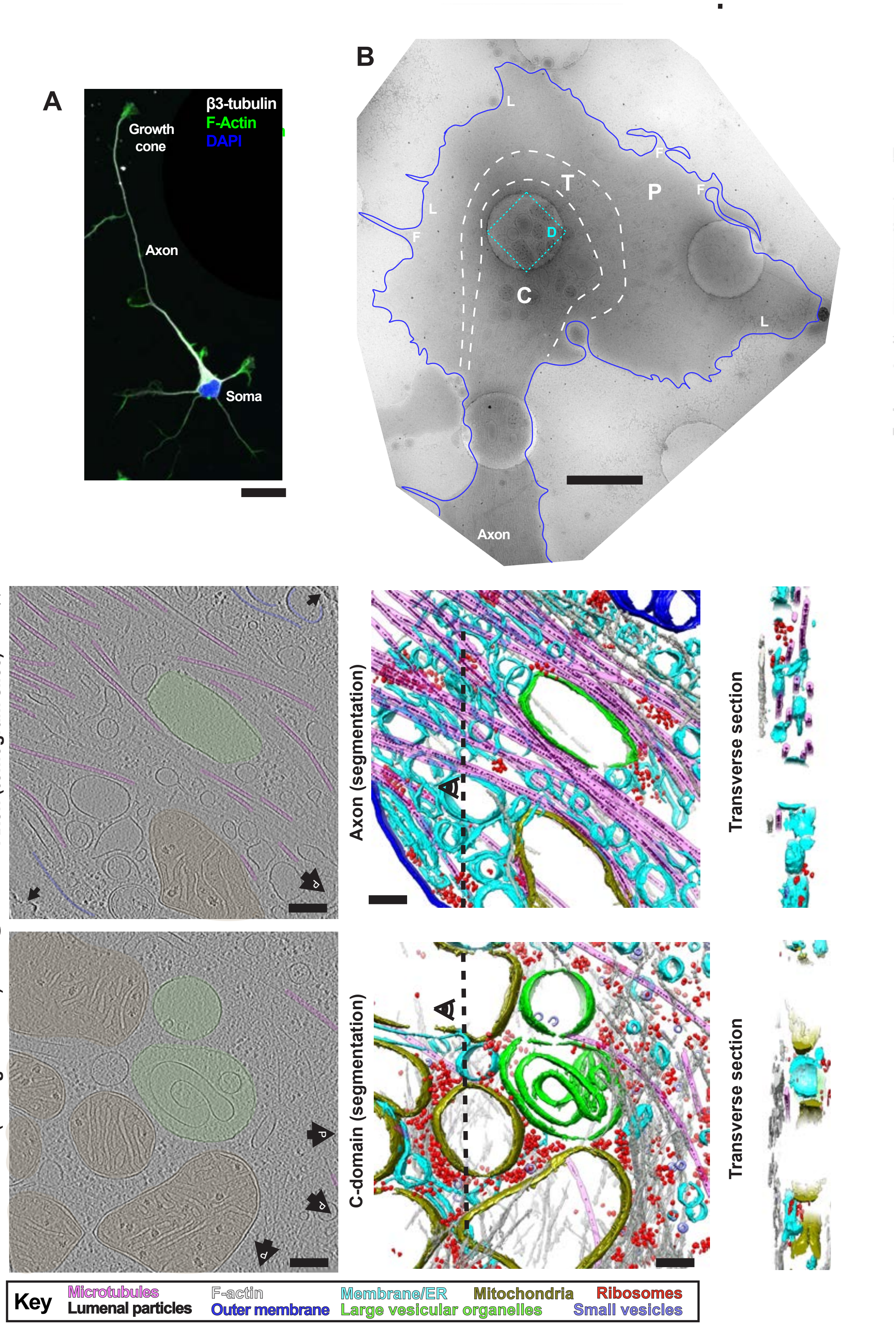
A
β3-tubulin
F-Actin
DAPI
Growth cone
Axon
Soma
B
L
F
T
P
D
C
Axon
C
Axon (tomogram slice)
Axon (segmentation)
Transverse section
D
C-domain (tomogram slice)
C-domain (segmentation)
Transverse section
Key
Microtubules
Lumenal particles
F-actin
Outer membrane
Membrane/ER
Large vesicular organelles
Mitochondria
Ribosomes
Small vesicles

Fig. S6. Cryo-electron tomography of primary neuronal growth cones and axons from Dcx KO mice.

(A) Mouse Dcx KO primary hippocampal neuron cultured for 3 days *in vitro*, demonstrating the characteristic polarised stage 3 morphology, labelled as indicated with DAPI (nucleus) and phalloidin (F-actin) dyes and β3-tubulin antibody. **(B)** Low magnification example cryo-EM image of a Dcx KO growth cone. The cell membrane is depicted in blue; the C-domain **(C)**, T-zone (T) and P-domain (P) are indicated; F, filopodia; L, Lamellipodia; H, underlying holes in the carbon substrate; I, Ice contamination; a representative region for tilt series data collection is indicated by the dotted turquoise boxed region. **(C, D)** Example tomogram and corresponding segmentation from a Dcx KO axon (C), C-domain (D). Left panel shows central section through a binned x 4 tomogram, with MTs (magenta), large vesicular organelles (green) and outer membrane (blue) indicated with overlaid false colouring. Direction of the leading edge is indicated with large black arrows containing a ‘P’ and edges of the carbon substrate hole are indicated with small black arrows. The middle panels show the corresponding whole volume 3D semiautomated segmentations. The right panels show a transverse section of the segmented region indicated with a dashed line in the corresponding central panel (section viewing direction also indicated in central panel). Segmentation false colouring is according to the key. Scale bars: A = 20 μm, B = 2 μm, C, D = 200 nm.

Journal of Cell Science • Supplementary information

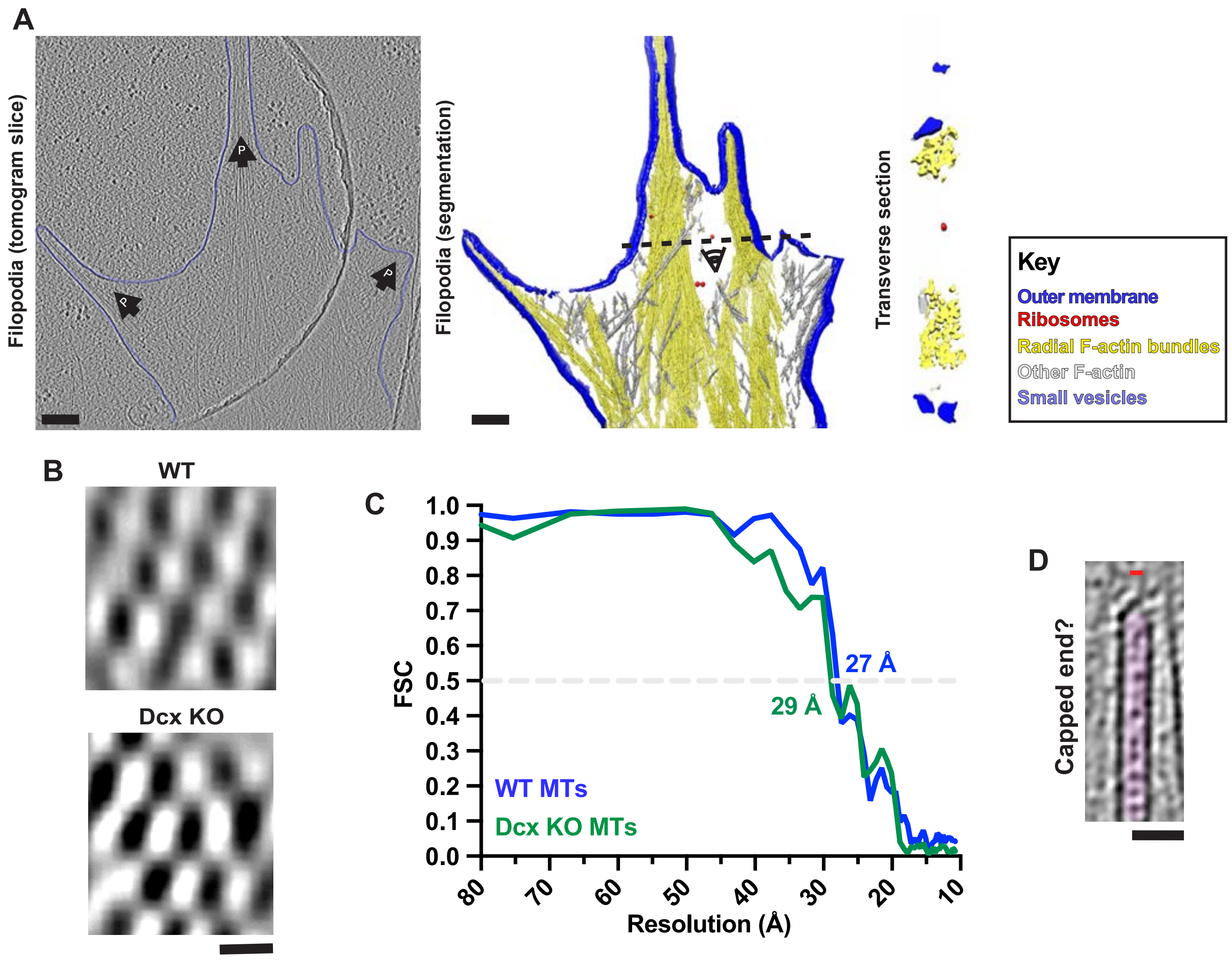
A
Filopodia (tomogram slice)
Filopodia (segmentation)
Transverse section
Key
Outer membrane
Ribosomes
Radial F-actin bundles
Other F-actin
Small vesicles
B
WT
Dcx KO
C
FSC
Resolution (Å)
27 Å
29 Å
WT MTs
Dcx KO MTs
D
Capped end?

Fig. S7. Normal ultrastructural organisation of filopodial Factin networks in Dcx KO growth cones.

(A) Example tomogram and corresponding segmentation from filopodia in a Dcx KO P-domain. Left panel shows central section through a binned x 4 tomogram, with outer membrane (blue) indicated with overlaid false colouring. Direction of the leading edge is indicated with large black arrows containing a ‘P’. The middle panel shows the corresponding whole volume 3D semi-automated segmentation. The right panel shows a transverse section of the segmented region indicated with a dashed line in the corresponding central panel (section viewing direction also indicated in central panel). Segmentation false colouring is according to the key. **(B)** Transverse views through filopodial F-actin bundles (~20 nm depth) in WT and Dcx KO Pdomains, showing their similar characteristic hexagonal arrangement. **(C)** Fourier Shell Correlation (FSC) curves generated from half sets of subtomogram-averages of MTs from WT (blue) and Dcx KO (green) neurons. Resolutions are indicated at the FSC = 0.5 criterion. **(D)** Longitudinal view of a potential capped MT minus end in a Dcx KO neuron, shown as a 10 nm thick slice through a 4x binned tomogram. The MT is false coloured in semi-transparent magenta. The minus end is indicated with a red ‘–’. Scale bars: A = 200 nm, B = 10 nm, C = 50 nm.

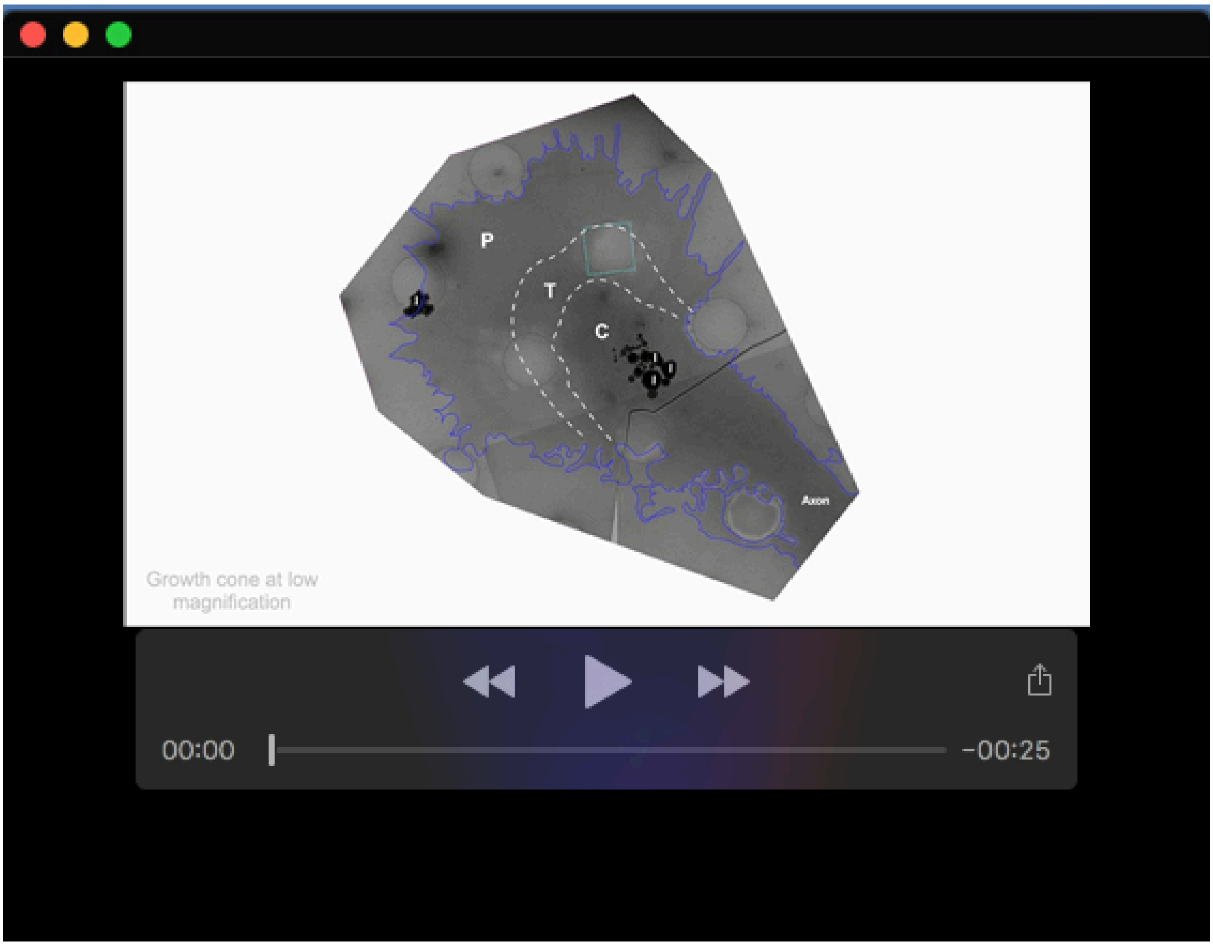

Movie 1. Overview of neuronal growth cone cryo-electron tomography data and segmentation.

Overview of cryo-ET procedures used during this study. The video starts with a low-magnification electron micrograph of a neuronal growth cone, with the cell membrane depicted in blue; the C-domain (C), transition zone (T) and peripheral domain (P) and ice contamination (I) indicated. A representative region over a hole in the carbon for tilt series data collection is indicated by the dashed cyan box. The video then shows the corresponding tilt series acquired for this region, with the peripheral leading edge of the growth cone indicated. Next, the corresponding reconstructed 2x binned tomogram is shown, followed by the corresponding semi-automated segmentation with features coloured as indicated.

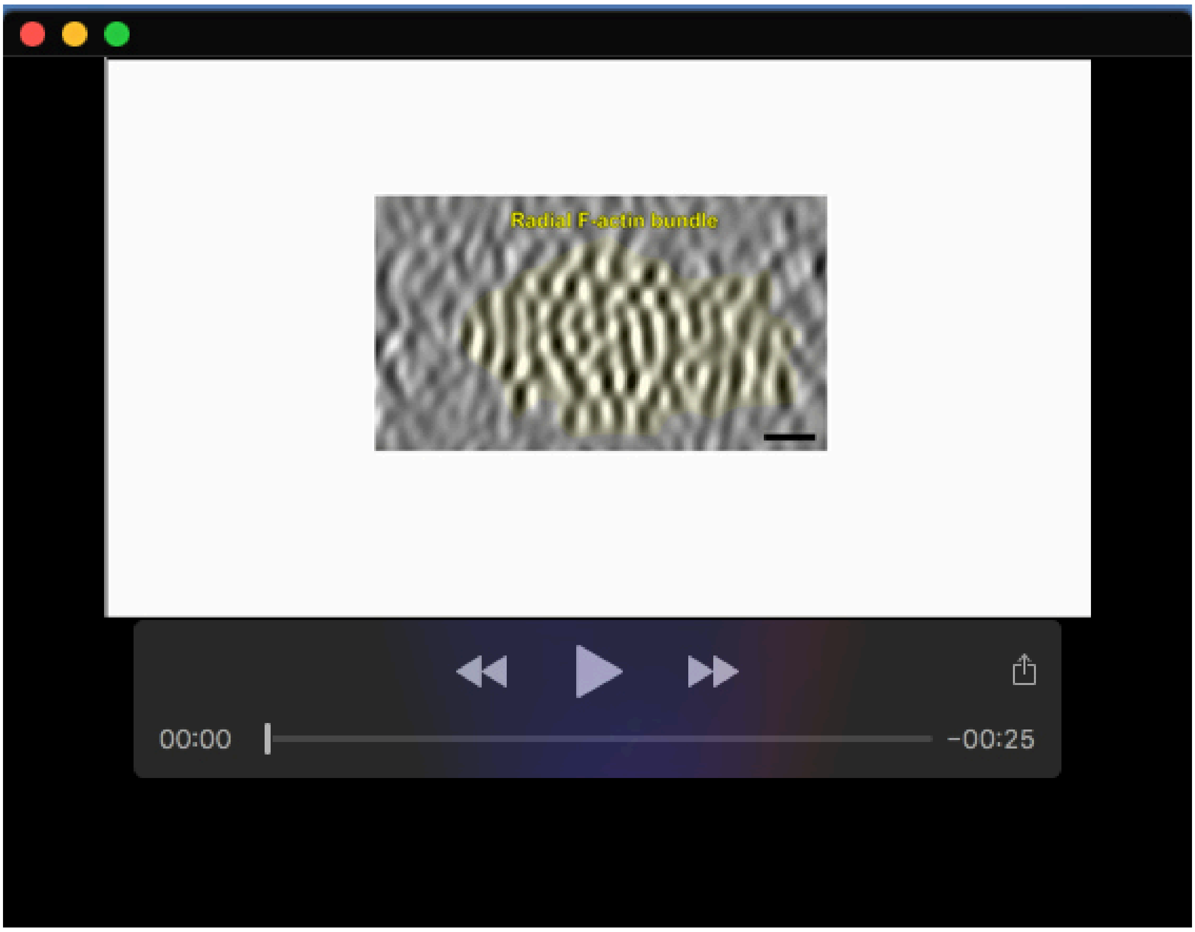

Movie 2. Fluidity within radial F-actin bundles.

Views though a radial F-actin bundle (as indicated in yellow false colour) in transverse sections (~20 nm depth), showing filaments in a rough hexagonal stacking, yet with a degree of fluidity with individual filaments being lost, added or moving position within the bundle. Scale bar = 25 nm.

Journal of Cell Science • Supplementary information

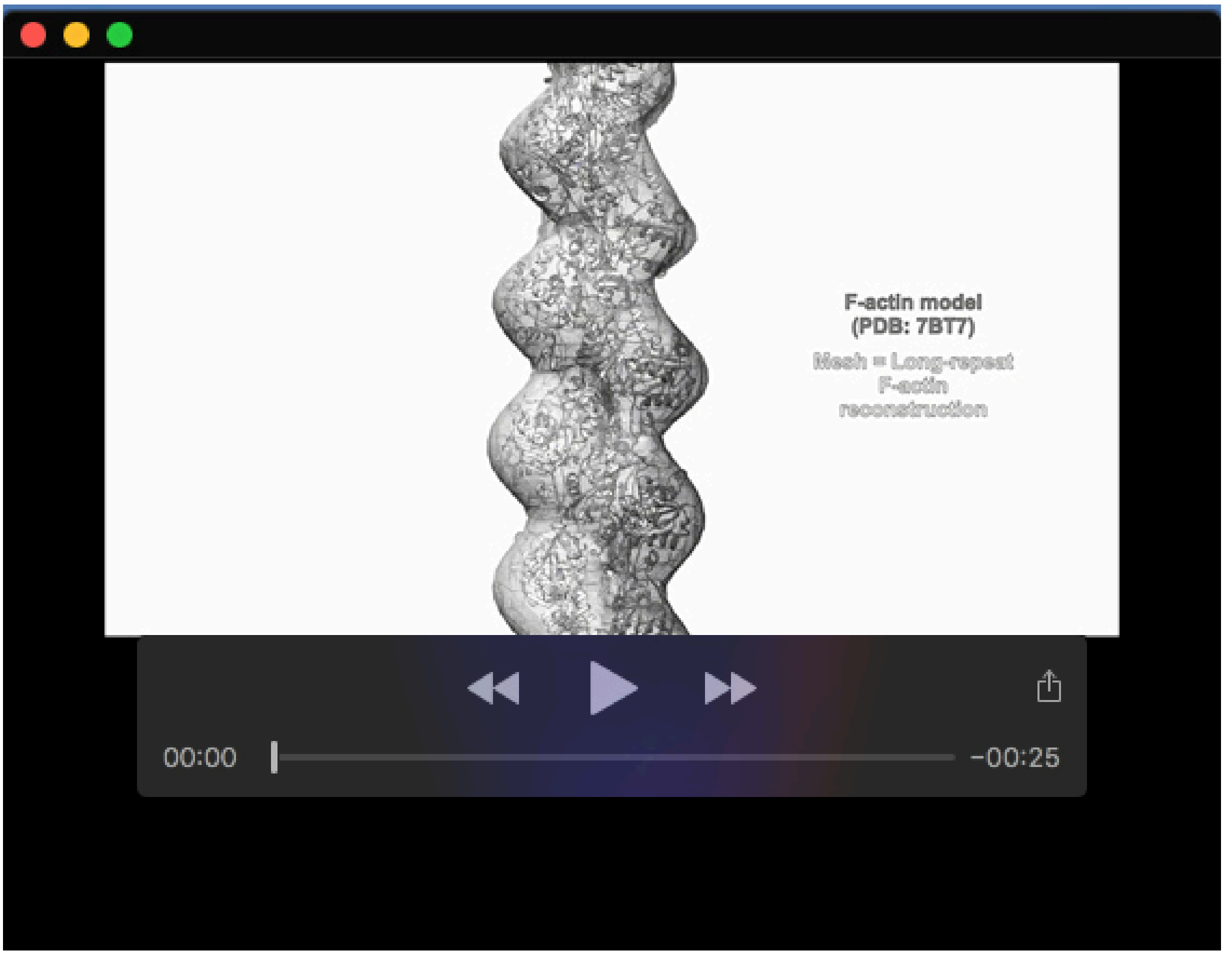

Movie 3. Sub-tomogram averages of long and short-repeat F-actin filaments within growth cones.

3D sub-tomogram averages calculated from volumes of long and short-repeat F-actin and fitted models of F-actin (PDB: 7BT7, (Kumari et al., 2020)) or F-actin-cofilin (PDB: 3JOS, (Galkin et al., 2011)), as indicated. The video starts with the F-actin model fitted into the long-repeat F-actin sub-tomogram average, showing a good match. The F-actin model is then swapped for the F-actin-cofilin model, that shows a poor match, with no density accounting for cofilin subunits. The F-actin sub-tomogram average is then swapped for the short-repeat F-actin sub-tomogram average, showing a good match, with extra density where cofilin binds. Mesh density is coloured to match within 10 Å of the underlying atomic models.

Journal of Cell Science • Supplementary information

REFERENCES

Galkin, V.E., A. Orlova, D.S. Kudryashov, A. Solodukhin, E. Reisler, G.F. Schroder, and E.H. Egelman. 2011. Remodeling of actin filaments by ADF/cofilin proteins. *Proc Natl Acad Sci U S A*. 108:20568-20572.

Kumari, A., S. Kesarwani, M.G. Javoor, K.R. Vinothkumar, and M. Sirajuddin. 2020. Structural insights into actin filament recognition by commonly used cellular actin markers. *EMBO J*. 39:e104006.

Lord, S.J., K.B. Velle, R.D. Mullins, and L.K. Fritz-Laylin. 2020. SuperPlots: Communicating reproducibility and variability in cell biology. *J Cell Biol* 219: e202001064.
