# Supplementary material for: Visualising the cytoskeletal machinery in neuronal growth cones using cryo-electron tomography
Source: J Cell Sci. 2022 Apr 8;135(7):jcs259234. doi: 10.1242/jcs.259234 (PMC9016625; doi:10.1242/jcs.259234)
Supplement: Reviewer comments [file joces-135-259234_review_history.pdf]

Journal of Cell Science

Visualising the cytoskeletal machinery in neuronal growth cones using cryo-electron tomography

Joseph Atherton, Melissa Stouffer, Fiona Francis and Carolyn A. Moores
DOI: 10.1242/jcs.259234

Editor: Giampietro Schiavo

Review timeline

Original submission:	12 August 2021
Editorial decision:	6 October 2021
First revision received:	21 December 2021
Editorial decision:	11 February 2022
Second revision received:	25 February 2022
Accepted:	2 March 2022

Original submission

First decision letter

MS ID#: JOCES/2021/259234

MS TITLE: Visualising the cytoskeletal machinery in neuronal growth cones using cryo-electron tomography

AUTHORS: Joseph Atherton, Melissa Stouffer, Fiona Francis, and Carolyn A Moores
ARTICLE TYPE: Research Article

We have now reached a decision on the above manuscript.

To see the reviewers' reports and a copy of this decision letter, please go to: https://submit-jcs.biologists.org and click on the 'Manuscripts with Decisions' queue in the Author Area. (Corresponding author only has access to reviews.)

As you will see, the reviewers raise a number of substantial criticisms that prevent me from accepting the paper at this stage. They suggest, however, that a revised version might prove acceptable, if you can address their concerns. If you think that you can deal satisfactorily with the criticisms on revision, I would be pleased to see a revised manuscript. We would then return it to the reviewers.

We are aware that you may be experiencing disruption to the normal running of your lab that makes experimental revisions challenging. If it would be helpful, we encourage you to contact us to discuss your revision in greater detail. Please send us a point-by-point response indicating where you are able to address concerns raised (either experimentally or by changes to the text) and where you will not be able to do so within the normal timeframe of a revision. We will then provide further guidance. Please also note that we are happy to extend revision timeframes as necessary.

Please ensure that you clearly highlight all changes made in the revised manuscript. Please avoid using 'Tracked changes' in Word files as these are lost in PDF conversion.

© 2022. Published by The Company of Biologists under the terms of the Creative Commons Attribution License (https://creativecommons.org/licenses/by/4.0/).

I should be grateful if you would also provide a point-by-point response detailing how you have dealt with the points raised by the reviewers in the 'Response to Reviewers' box. Please attend to all of the reviewers' comments. If you do not agree with any of their criticisms or suggestions please explain clearly why this is so.

Reviewer 1

Advance summary and potential significance to field

This paper is a first-class piece of structural cell biology. The authors have used excellent methods for the study of cellular fine structure to examine the part of a neuron that is most interesting for its ability to extend long process and develop connections. The methods used for mouse construction and maintenance are all good, as are those for cell culture and the preparation of samples for cryoEM. The instrumentation employed is excellent, and the tomograms presented are quite beautiful. Methods for segmentation of cytoplasmic structures are fine, although nothing seems to have been done to evaluate their accuracy, and the analysis of the structural data is both intelligent and thorough. The paper is largely descriptive, but it is description of a high order and the resulting data will serve as benchmarks for many future papers in which perturbations other than simply the doublecortin deletion describe here are used and studied. For all these reasons, I strongly support the publication of this work.

Comments for the author

Some of the writing of the paper itself is a bit loose. While reading the MS to write this review, I kept a running list of issues that came to mind, as shown in Specific Comments below. I encourage the authors to use these musings from an old fan to tighten their paper and make it more interesting.
Abstract. Yes, neurons do move, but this is not growth cone dependent, and therefore not part of this paper.
Ln 8 Transition zone has not yet been defined.
Sup fig 1A The blue of the DAPI is invisible on my screen. Can you brighten it? Fig 1d, don't you mean a slice from a tomogram? There is only one large black arrow; remove plural.
P3, para2, ln 3, Saying that the axon shaft "precedes" the growth cone seems backwards. Is there a reason to state it that way? From a physiological point of view, the GC leads!
Ln 5, the sentence beginning "The axon connects…" runs on. Break it into two.
P4, para2, ln 2. It is probably a misconception to think about "the precise order" of a region of a cell. Structure is almost never precise at a cellular level. For example, if you looked at 5 growth cones and asked, how similar they are to one another, it would be only at an overall level, not at a precise level. This wording is misleading.
General comment on this paragraph. The words chosen to describe what it not known are all associated with function: actin disassembly, actin arcs generate T-zone, MTs influencing growth cones, for examples. Careful description of static structures, as in cryoET, are not likely to answer dynamic questions, so this paragraph introduces false expectations.
Video 1 is really excellent.
Fig 2 legend, 2A Now there are two black arrows and you refer to them as one.
P5 para3 Did you do anything to assess the validity of your "semi automatic" segmentation protocol? Can you say somewhere how valid it is likely to be? The pictures are pretty, but what was missed, what was falsely included, and how close are the graphic surfaces to the membranes or fibers they represent?
P6 para3: the discussion of the hexagonal actin bundles. The bundles are not perfect hexagons, but are elongated in the direction of the electron beam. It seems likely that this is a computational distortion, resulting from the missing wedge of data that is intrinsic to single tilt axial tomography. I believe you could assess this likelihood from information about asymmetric point spread in the literature and it would clarify a point about the real packing geometry of the actin.
P7, ln 7 and following. It would be helpful to note the similarity (and/or differences) between the dimensions of your actin filaments and bundles with those described elsewhere.
Fig. 3 H. The long-pitch fits of actin's atomic structure to the cryoET envelope looks beautiful, but I wonder how much the displayed fit could be altered and still look like a good fit? The EM density distributions are very smooth, so I can imagine a good bit of sloshing around would be possible.

© 2022. Published by The Company of Biologists under the terms of the Creative Commons Attribution License (https://creativecommons.org/licenses/by/4.0/).

P9 para3, last sentence. This statement about "regulatory proteins" seems a bit much. The paper presents evidence for cofilin on the long-period actin bundles, but thus far that's it. The later evidence on doublecourtin show surprisingly little structural alteration, given the dire consequences of the mutation.
P8, last paragraph: The observation of 141 MTs from 11 tomograms showing exclusively 13 protofilament organization is significant. A recent preprint from the Chretien lab (Guyomar et al., doi: https://doi.org/10.1101/2021.07.14.452321) describes extensive variation in the lattice of even one MT. Their finding that this pleiotropy is less for MT assembled from frog egg extracts implies that the observed plasticity of the MT lattice might be an artifact of MTs assembled in vitro from brain tubulin, a notoriously complex mixture of proteins. The evidence presented here supports that interpretation and may help to steer the field away from a lot of nonsense about flaccid tubulin lattices. I hope the authors will cite the new work and make a point of their own observations.
P10 para 2 and Fig 5E: These ends certainly do look blunt, but I wonder if the authors have used rotary sampling of these ends to see if any of the protofilaments flares out from the MT axis at some different orientation from that shown? There are nice flares in F and 8E (for a possible guide to terminology describing MT ends, see Gudimchuk and McIntosh, https://doi.org/10.1038/s41580-021-00399-x). This mode of viewing is easy to do, and it might be informative.
Fig. 8H shows one datum in the KO C-domain that is a real outlier. Is this point the origin of the statistically significant difference? If so, it's a bit dubious, because one point may be an outlier for various reasons, such as preparation artifact. Can this possibility be dismissed?
Methods P17 Fluorescence microscopy. Please define PEM buffer.

Reviewer 2

Advance summary and potential significance to field

Atherton et al. studied neuronal growth cone using cryo-electron tomography.The authors found some interesting aspects of cytoskeleton architecture organization that may be of interest to the community. However, I have some technical reservations that the authors may want to address before the manuscript is ready for publication.

Comments for the author

Major points
1. To my understanding figure 2D was manipulated. The authors inserted fading circles instead of showing the real data as they indicated in the figure legend, moreover, the authors write that Fig. 2D is the same as shown in Fig. 2Biii (D) . The latter shows a distorted arrangement while Fig. 2D shows perfect symmetry. In any case, a central density surrounded by 6 densities is not an hexagonal crystal packing.
2. The authors do not consider the effect of the missing-wedge and its influence on interactions which are not on the x-y plane. Actin filaments are elongated along the Z-axis of their tomograms, therefore the "diagonal" bridges detected may cause by it- as actin are artificially close to each other. Even if there are some densities emanated toward actin filaments at a different level within the thickness of the cell, these densities will also be artificially elongated. In summary, reporting interactions along the missing wedge requires additional experiments, e.g. modeling.

3. The averaged structures of actin and MTs indicated a resolution of 2.7-3.1nm. This is a very low resolution for CTF corrected tomogram. In fact, some of the data would exhibit information in higher resolution even without CTF correction (-3um gives ~2.4 nm ,1st destructive interference of the beam). The authors acquired their data using dose symmetric scheme, which developed to allow high resolution structural determination using sub-tomogram averaging.
Recently, several studies indicated much higher actin and MT resolved structure from similar quality of cellular data.

4. The resolution reported here for F-actin is not high-enough to distinguish between the barbed and pointed actin-end (~20A) . Therefore, it is not clear how the authors can dock unambiguously actin structure into their maps.

© 2022. Published by The Company of Biologists under the terms of the Creative Commons Attribution License (https://creativecommons.org/licenses/by/4.0/).

Other points:
Figure. 2E, the authors draw yellow lines on top of the real density of actin.
The reader should see the data and not the artificial drawings.
Page 6 and Figure 2E. The authors found a repeating crosslinking every 37nm. If this is the case, 2D averages of the crosslinkers can be conducted and indicate how statistically relevant this finding.
Figure 3B shows hollow structures. A section through the averaged structure (gray value densities) would be more relevant. The packing shows different dimensions than in Fig. 2D
The authors use the term pitch for actin repeat. However, the pitch and repeat are identical only when the pitch in an integer number of subunits. This is not the case for actin filaments.
None of the structures nor tomograms were submitted to the EMBD/EMPAIR bank.

Reviewer 3

Advance summary and potential significance to field

In the manuscript "Cryo-ET of neuronal growth cones" the authors aim at characterizing the mouse hippocampal neuron growth cones' 3D nanoarchitecture in its "central domains (C), transition zones (T) and peripheral domains (P) " as well as their interface with axons through the use of cryo-ET and machine learning-based segmentation . The authors employ segmentation analysis to quantitatively assess the molecular distribution of cellular components within these different regions of WT growth cones and those of doublecortin Dcx knockout neurons (Dcx in mouse), a regulator of both growth cone F-actin and MT organization. The overall aim here is providing insights allowing unraveling the molecular basis for growth cone cytoskeleton regulation and coordination. A timely and exciting study.

Comments for the author

A few suggestions and queries:
The cryo-EM samples were unfixed and plunge-frozen whereas the LM data was acquired on fixed cells with the MTs stabilized by taxol. Can the authors provide evidence that the preparations are equivalent on the LM as well as cryo-EM level?
It is unclear how the identification/boundaries of the domains were established.
To avert the impression of macro level designation, the authors need to expand.
Also, why the Authors opt to acquire data exclusively on regions that are over the 2 μm diameter holes in the carbon. It will be informative to compare regions nearby on carbon associated with the same domains, as well as regions between domains while on carbon. Jasmin et al, 2016 suggested holes induce changes in the cytoskeleton of certain cells and the authors need to discuss and convince the reader that this is not an issue in the current study.
Concerning the effect of the missing wedge, the authors need to explain why they believe that the rotational symmetry of the holes ensures that their conclusions are "not significantly affected" by the potential attenuation of filament signals.
The statement, as it stands, is unclear.
The authors mentioned that neural network-driven density segmentation provided a quantitative analysis as well as absolute segmentation volumes were measured in Chimera. Can the authors expand on this topic? The validity of the quantification will depend on the quality of the NN segmentation, which, in turn, is not well defined and depends on how the user trains the network. To test and determine consistency, the authors should consider having at least three blinded and independent repeats of the NN segmentation and subsequent quantification by three different users and then report the statistics. Otherwise, the authors should remove any reference to "quantitative".
Fascin: the authors are inclined to suggest that the architecture of P-domain Actin bundles is most consistent with F-actin-Fascin crosslinks. This statement should be quantified, currently it is speculative and based on literature, not even measurements. Again, statistics of the measurements should be compiled and reported.
Cofilin-bound actin filaments exist in the P-domain: Rigid-body fitted F-actin-cofilin exhibited a 0.87 model to map cross-correlation (CC) calculated in Chimera. The authors claim that this constitutes a "very good fit" is unconvincing. The value of the CC is highly sensitive

© 2022. Published by The Company of Biologists under the terms of the Creative Commons Attribution License (https://creativecommons.org/licenses/by/4.0/).

to resolution, noise level and scaling and an absolute value is not meaningful. If the authors want to argue that it is more likely that these filaments contain cofilin than not, they need to conduct a comparative analysis where they compare fitting of bare actin (with adjusted pitch), fitting of actin with at least one alternative binding partner
(eg dystrophin; again with adjusted pitch), and the fitting of actin-cofilin. This needs to be performed on at least three independent subsets of the data so statistics can be compiled. Then, the authors need to show that the statistics indicate actin-cofilin fits significantly better than bare actin and the alternative binder. Alternatively, the authors can remove the statement about the quality of the fit (including the value) and clarify in the text that their analysis is only qualitative and possible alternatives (including bare actin with modified twist) exist.
Next the authors opt to capture tomograms within holes, can the authors quantify the ratio of observable filament detached from bundles when it does not occur at the hole edges? Holes, perforated surfaces were shown previously to affect actin nanoscale organization (Jasmin et al, 2016).
Arp2/3 mediated branches in the P-domain: Branching seems to be identified emanating from the sides of actin filaments, with ~ 70 degrees angels, were the angles measured, how many of them, and what were their SD?
Close association of MTs with F-actin seems to be casual, as this observation is of key importance the authors, it needs to be quantified in 3D.

First revision

Author response to reviewers' comments

Reviewer 1 Advance Summary and Potential Significance to Field:

This paper is a first-class piece of structural cell biology. The authors have used excellent methods for the study of cellular fine structure to examine the part of a neuron that is most interesting for its ability to extend long process and develop connections. The methods used for mouse construction and maintenance are all good, as are those for cell culture and the preparation of samples for cryoEM. The instrumentation employed is excellent, and the tomograms presented are quite beautiful. Methods for segmentation of cytoplasmic structures are fine, although nothing seems to have been done to evaluate their accuracy, and the analysis of the structural data is both intelligent and thorough. The paper is largely descriptive, but it is description of a high order and the resulting data will serve as benchmarks for many future papers in which perturbations other than simply the doublecortin deletion describe here are used and studied. For all these reasons, I strongly support the publication of this work.

Reviewer 1 Comments for the Author:
Some of the writing of the paper itself is a bit loose. While reading the MS to write this review, I kept a running list of issues that came to mind, as shown in Specific Comments below. I encourage the authors to use these musings from an old fan to tighten their paper and make it more interesting.

1. Abstract. Yes, neurons do move, but this is not growth cone dependent, and therefore not part of this paper.
We have edited the text accordingly.

2. Ln 8 Transition zone has not yet been defined.
We have edited the text accordingly.

3. Sup fig 1A The blue of the DAPI is invisible on my screen. Can you brighten it?
Sup Fig 1A has been updated to make the DAPI staining clearer.

4. [Sup] Fig 1d, don't you mean a slice from a tomogram?
Yes - we have corrected the text.

© 2022. Published by The Company of Biologists under the terms of the Creative Commons Attribution License (https://creativecommons.org/licenses/by/4.0/).

5. There is only one large black arrow; remove plural.
We have corrected the text.

6. P3, para2, ln 3, Saying that the axon shaft “precedes” the growth cone seems backwards. Is there a reason to state it that way? From a physiological point of view, the GC leads!
We have edited the text accordingly.

7. Ln 5, the sentence beginning “The axon connects…” runs on. Break it into two.
We have edited the text accordingly.

8. P4, para2, ln 2. It is probably a misconception to think about “the precise order” of a region of a cell. Structure is almost never precise at a cellular level. For example, if you looked at 5 growth cones and asked, how similar they are to one another, it would be only at an overall level, not at a precise level. This wording is misleading.
This is a good point – we have changed the text, deleting “precise”.

9. General comment on this paragraph. The words chosen to describe what it not known are all associated with function: actin disassembly, actin arcs generate T-zone, MTs influencing growth cones, for examples. Careful description of static structures, as in cryoET, are not likely to answer dynamic questions, so this paragraph introduces false expectations.
We agree that we should be clear about what can be expected from our cryo-ET data. Nevertheless, they do provide informative snapshots of dynamic processes – e.g. the direct observation of 27nm repeat, cofilin-like decorated actin filaments is indicative of the mechanism by which actin bundles are disassembled. We have edited this paragraph to manage the reader’s expectations concerning our data.

10. Video 1 is really excellent.
Many thanks!

11. Fig 2 legend, 2A Now there are two black arrows and you refer to them as one.
We have corrected the text accordingly.

12. P5 para3 Did you do anything to assess the validity of your “semi automatic” segmentation protocol? Can you say somewhere how valid it is likely to be? The pictures are pretty, but what was missed, what was falsely included, and how close are the graphic surfaces to the membranes or fibers they represent?
This is a fair point - Reviewer 3 asked something similar (point 35). To address these questions, we have now expanded the relevant Methods text (p. 19) and have added a Supplementary Figure (new Sup. Fig. 2) to illustrate the steps involved in segmentation, which include manual quality control interventions. In overview, while the automated aspects of our approach support efficient and non-biased segmentation, the subsequent manual interventions reduce false positives. The manual steps inevitably reduce our data throughput but still allow efficient annotation of relatively large cell volumes. Our study focuses on a subset of better characterised molecular features which further increases confidence in this approach.

13. P6 para3: the discussion of the hexagonal actin bundles. The bundles are not perfect hexagons, but are elongated in the direction of the electron beam. It seems likely that this is a computational distortion, resulting from the missing wedge of data that is intrinsic to single tilt axial tomography. I believe you could assess this likelihood from information about asymmetric point spread in the literature and it would clarify a point about the real packing geometry of the actin.
We thank the reviewer (and the other reviewers (point 22 and 34) for highlighting this aspect of our analysis that we hadn’t sufficiently considered. The reviewer is absolutely correct concerning the effect of the missing wedge on the actin bundle reconstructions. Having established that the missing wedge is indeed affecting our data, we have revised the main text (p. 7) and relevant figure panels (Fig. 3) accordingly. As the reviewer notes, this allows us to clarify a point about the packing geometry of the actin and flag the challenges of determining actin crosslinking protein distribution, also seen elsewhere in the literature (e.g. Aramaki et al, 2016).

14. P7, ln 7 and following. It would be helpful to note the similarity (and/or differences) between

© 2022. Published by The Company of Biologists under the terms of the Creative Commons Attribution License (https://creativecommons.org/licenses/by/4.0/).

the dimensions of your actin filaments and bundles with those described elsewhere.
This is an important point. Given the intrinsic complexity of our data, we have opted to describe it without wider comparison in the Results section for clarity of presentation. However, we included several points of comparison to other work in the Discussion (particularly p13) and have further elaborated on this in response to this comment.

15. Fig. 3 H. The long-pitch fits of actin's atomic structure to the cryoET envelope looks beautiful, but I wonder how much the displayed fit could be altered and still look like a good fit? The EM density distributions are very smooth, so I can imagine a good bit of sloshing around would be possible.
The reviewer is correct that the actin sub-tomogram averages are not sufficiently featureful to provide information about the fit of the F-actin models beyond differentiation of the subunits. However, our interpretations do not depend on the precision of our model fitting. Rather, the F-actin model aids comparison with the short-pitch filament population and highlights the extra density bound to them. We hope this is clearest in Supplementary Video 3. We continue to think this analysis is useful and have therefore retained it. However, in response to this comment and that of the other reviewers (point 24 and 37) we have adjusted the text on p8 to more clearly indicate the limits of interpretability of the fitting.

16. P9 para3, last sentence. This statement about "regulatory proteins" seems a bit much. The paper presents evidence for cofilin on the long-period actin bundles, but thus far that's it. The later evidence on doublecourtin show surprisingly little structural alteration, given the dire consequences of the mutation.
This is a fair point – we have deleted the second part of this sentence.

17. P8, last paragraph: The observation of 141 MTs from 11 tomograms showing exclusively 13 protofilament organization is significant. A recent preprint from the Chretien lab (Guyomar et al., doi: https://doi.org/10.1101/2021.07.14.452321) describes extensive variation in the lattice of even one MT. Their finding that this pleiotropy is less for MT assembled from frog egg extracts implies that the observed plasticity of the MT lattice might be an artifact of MTs assembled in vitro from brain tubulin, a notoriously complex mixture of proteins. The evidence presented here supports that interpretation and may help to steer the field away from a lot of nonsense about flaccid tubulin lattices. I hope the authors will cite the new work and make a point of their own observations.
Thanks to the reviewer for highlighting this aspect of our data – we have added text in the discussion (p15) to amplify this point.

18. P10 para 2 and Fig 5E: These ends certainly do look blunt, but I wonder if the authors have used rotary sampling of these ends to see if any of the protofilaments flares out from the MT axis at some different orientation from that shown? There are nice flares in F and 8E (for a possible guide to terminology describing MT ends, see Gudimchuk and McIntosh, https://doi.org/10.1038/s41580-021-00399-x). This mode of viewing is easy to do, and it might be informative.
We thank the reviewer for this suggestion. We attempted the mentioned rotary sampling of the MT ends but found that our single tilt, cellular data are too noisy and distorted by the missing wedge to reliably trace individual protofilaments. As an example, in Figure 1 for Reviewers, we show first, a ~160nm deep section through an MT, in which the direction of the missing wedge is indicated. We then show 8nm deep, 1.6nm spaced transverse sections towards the end of this MT, which illustrate how the noisiness of the cellular data make protofilament tracking unreliable. In the future, we hope that improvements in data collection and new developments in cryo-ET software, can improve the quality of the data such that we can conduct this analysis. Thanks also for the recommendation about end nomenclature summarised by Gudimchuk & McIntosh – we have adjusted the text on pages 9-10 and in the Discussion accordingly with the intention of being more clear about our end structure categories.

© 2022. Published by The Company of Biologists under the terms of the Creative Commons Attribution License (https://creativecommons.org/licenses/by/4.0/).

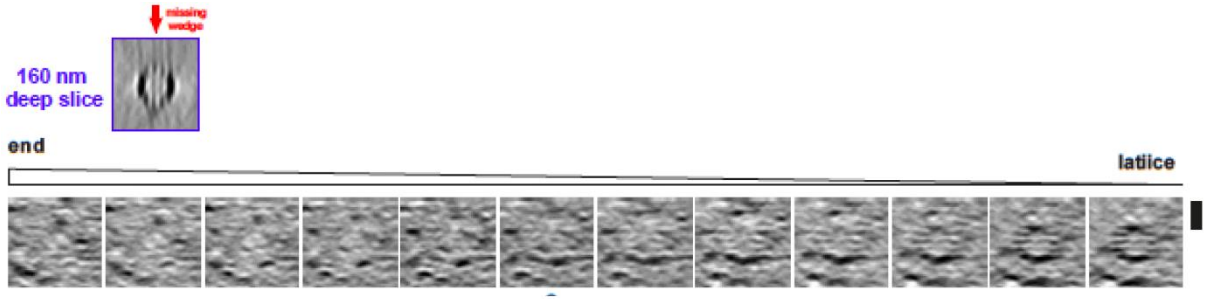

Figure 1 for Reviewers. Scale bar = 25 nm

19. Fig. 8H shows one datum in the KO C-domain that is a real outlier. Is this point the origin of the statistically significant difference? If so, it's a bit dubious, because one point may be an outlier for various reasons, such as preparation artifact. Can this possibility be dismissed?
This is a fair point – thanks to the reviewer for flagging this limitation of our small dataset. We repeated the analysis without the outlier and the differences are not significant ($p = 0.1083$, Mann Whitney) between WT and KO. For complete transparency, we have added this analysis and flagged that we cannot exclude that the outlier could have been caused by a number of factors including preparation artifact (p12 and figure legend). We have also removed relevant text in the Discussion (p16), noting that there are other strands of evidence to support disruption of MT organisation in the KO neurons

20. Methods - P17 Fluorescence microscopy. Please define PEM buffer.
Apologies – we have added this definition.

Reviewer 2 Advance Summary and Potential Significance to Field:
Atherton et al. studied neuronal growth cone using cryo-electron tomography. The authors found some interesting aspects of cytoskeleton architecture organization that may be of interest to the community. However, I have some technical reservations that the authors may want to address before the manuscript is ready for publication.

Reviewer 2 Comments for the Author:

Major points

21. 1. To my understanding figure 2D was manipulated. The authors inserted fading circles instead of showing the real data as they indicated in the figure legend, moreover, the authors write that Fig. 2D is the same as shown in Fig. 2Biii (D). The latter shows a distorted arrangement while Fig. 2D shows perfect symmetry. In any case, a central density surrounded by 6 densities is not an hexagonal crystal packing.
First, we state categorically that Fig. 2D in our original submission was not manipulated, although we had added some false (yellow) colour to facilitate reader comprehension. By including the label 'D' in the original version of panel Fig.2Biii, we had meant to indicate, for the purposes of comparison with Fig. 2D, the repeating hexagonal unit in the context of a larger bundle. However, because the images are of different actin bundles, we can see that this was confusing without full explanation. To address the reviewer's concerns, we have therefore:
i) removed the false colouring from Fig. 2D
ii) removed the label 'D' from panel 2Biii
iii) more clearly explained how panels 2Biii and 2D relate to each other in the legend
iv) replaced references to hexagonal crystal packing to hexagonal array, which we accept is more accurate.
We have also added more information to Fig. 2D to address other aspects of these data, including an additional long axis measurement of the hexagonal packing.

22. 2. The authors do not consider the effect of the missing-wedge and its influence on interactions which are not on the x-y plane. Actin filaments are elongated along the Z-axis of their tomograms, therefore the "diagonal" bridges detected may cause by it- as actin are artificially

© 2022. Published by The Company of Biologists under the terms of the Creative Commons Attribution License (https://creativecommons.org/licenses/by/4.0/).

close to each other. Even if there are some densities emanated toward actin filaments at a different level within the thickness of the cell, these densities will also be artificially elongated. In summary, reporting interactions along the missing wedge requires additional experiments, e.g. modeling.

We thank the reviewer for pointing out this error in our approach in analysing the bundling data. Reviewer 3 made a similar point (point 34). Detailed investigation of our data confirmed that missing-wedge derived distortions were likely making significant contributions to density we originally assigned as diagonal cross-links (Figure 2 for Reviewers).

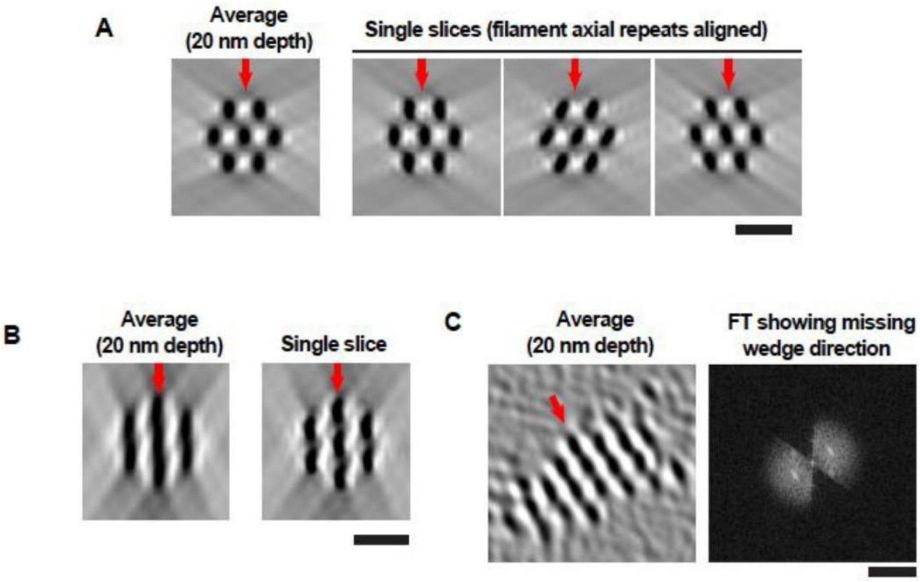

*Figure 2 for Reviewers. **A)** Density for a 7 F-actin hexagonal array with synchronised helical repeats was simulated at a resolution of 30 Å from F-actin models (PDB: 7BT7) using the Chimera 'molmap' command. To simulate the missing wedge effect in our tomograms reconstructed from tilt series of -60 to +60, a wedge shaped mask in Fourier space was applied to the density, using the EMAN2 processor mask.fft.wedge (Tang et al, 2007, PMID: 16859925). Transverse views of the resulting volume are shown after binning by 4x. The direction of the missing wedge is illustrated with a red arrow. The left panel depicts ~20 nm depth section, while the right-hand group of images are different single slices taken at points along the filament axis where the cylindrical shape of adjacent F-actin filaments align due to their synchronised helical repeats. **B)** The model was rotated 30° relative to that in panel A before applying the missing wedge effect, such that adjacent filaments align along the missing wedge. Slices were taken at a point along the filament axis where the cylindrical shape of adjacent F-actin filaments align. **C)** Example of significant artefacts produced by the missing wedge effect in our tomograms. The left hand panel is a transverse section of ~20 nm depth. The direction of the missing wedge for each image is illustrated with a red arrow and by the Fourier transform of each image shown to the right. Scale bars: A-C = 20 nm.*

We have therefore removed reference to them in the manuscript (p. 7 and Fig. 3B-D). Instead, we now focus on the relative arrangement of individual filaments within these bundles, which is reliably represented in the data and shows the ordered displacement of adjacent filaments by one subunit (Fig. 3B and Sup. Fig. 4 (former Sup. Fig. 3)). This arrangement is another reason why the originally performed sub-tomogram average did not accurately represent the underlying data.

23. 3. The averaged structures of actin and MTs indicated a resolution of 2.7-3.1nm. This is a very low resolution for CTF corrected tomogram. In fact, some of the data would exhibit information in higher resolution even without CTF correction (-3um gives ~2.4 nm ,1st destructive interference of the beam). The authors acquired their data using dose symmetric scheme, which developed to allow high resolution structural determination using sub-tomogram averaging. Recently, several studies indicated much higher actin and MT resolved structure from similar quality of cellular data.

The purpose of our study was to characterise the organisation of the growth cone cytoskeleton and

© 2022. Published by The Company of Biologists under the terms of the Creative Commons Attribution License (https://creativecommons.org/licenses/by/4.0/).

thereby shed light on its regulation. To do this, we prioritised larger cellular volumes, slightly lower magnification data collection, and therefore larger pixel size (5.38 Å per pixel) than is used in higher resolution studies. Because our data provide representative snapshots of growth cone regions, the cytoskeleton components are also overall heterogeneous - e.g. curved and/or irregularly bundled filaments - and therefore the subset of particles suitable for sub-tomogram averaging were also smaller than is typical of higher resolution studies. Even though the purpose of our experiment was not high resolution sub-tomogram averaging, we used dose symmetric tilt acquisition scheme and applied CTF correction to maximise the quality of our data.

24. 4. The resolution reported here for F-actin is not high-enough to distinguish between the barbed and pointed actin-end (~20A) . Therefore, it is not clear how the authors can dock unambiguously actin structure into their maps.
This is a fair point, and similar to a point made by reviewer 1 (point 15). As we commented in point 15, our interpretations do not depend on the precision of our model fitting. Rather, the F-actin model aids comparison with the short-pitch filament population and highlights the extra density bound to them. In response to the reviewers' comments, we have adjusted the text on p8 and the figure legend to more clearly indicate the limits of interpretability of the fitting.

Other points:
25. Figure. 2E, the authors draw yellow lines on top of the real density of actin. The reader should see the data and not the artificial drawings.
We originally included the false colouring to facilitate reader understanding but we have now removed it

26. Page 6 and Figure 2E. The authors found a repeating crosslinking every 37nm. If this is the case, 2D averages of the crosslinkers can be conducted and indicate how statistically relevant this finding.
We already provide statistical analysis of bundle spacing in the right-hand graph in panel 2E. Concerning the proposal to undertake 2D averaging of these crosslinkers, given the noted limitations of the data, our attempts to perform this did not produce anything convincing. However, we are also not sure we have caught the reviewer's point relating to statistical relevance and would be happy to receive further clarification.

27. Figure 3B shows hollow structures. A section through the averaged structure (gray value densities) would be more relevant.
In response to other comments from the reviewers, this depiction is no longer included in the figure.

28. The packing [in 3B] shows different dimensions than in Fig. 2D
Because of the noted limitations of the originally included sub-tomogram average, we have removed the depiction of it that was in the original Fig. 3B.

29. The authors use the term pitch for actin repeat. However, the pitch and repeat are identical only when the pitch in an integer number of subunits. This is not the case for actin filaments.
We have corrected the text accordingly.

30. None of the structures nor tomograms were submitted to the EMBD/EMPAIR bank.
Thank you for this suggestion – we are submitting exemplar tomograms from both WT and mutant neurons to EMPIAR. Given the limited resolution and docking ambiguity of the actin reconstructions, we do not think it is appropriate to deposit these structures in EMDB.

Reviewer 3 Advance Summary and Potential Significance to Field:
In the manuscript "Cryo-ET of neuronal growth cones" the authors aim at characterizing the mouse hippocampal neuron growth cones' 3D nanoarchitecture in its "central domains (C), transition zones (T) and peripheral domains (P) " as well as their interface with axons through the use of cryo-ET and machine learning-based segmentation . The authors employ segmentation analysis to quantitatively assess the molecular distribution of cellular components within these different regions of WT growth cones and those of doublecortin Dcx knockout neurons (Dcx in mouse), a regulator of both growth cone F-actin and MT organization. The overall aim here is providing

© 2022. Published by The Company of Biologists under the terms of the Creative Commons Attribution License (https://creativecommons.org/licenses/by/4.0/).

insights allowing unraveling the molecular basis for growth cone cytoskeleton regulation and coordination. A timely and exciting study.

Reviewer 3 Comments for the Author:
A few suggestions and queries:

31. The cryo-EM samples were unfixed and plunge-frozen whereas the LM data was acquired on fixed cells with the MTs stabilized by taxol. Can the authors provide evidence that the preparations are equivalent on the LM as well as cryo-EM level?
The purpose of the LM images in Sup Fig 1A,B is to provide a point of reference between the neuronal system used in our study and previous growth cones studies, the large majority of which have also used LM. These images provide confirmation of the overall growth cone organisation and also confirm native DCX localisation seen in prior work, as indicated by the relevant citations in the text (p5). The different types of preparations involved relate primarily to the speed of fixation for EM (extremely fast) and LM (slower) but we do not make direct correlations between our LM and EM data. This would require fluorescent labelling of endogenous DCX – this would be of great interest but we feel is well beyond the scope of our current study.

32. It is unclear how the identification/boundaries of the domains were established. To avert the impression of macro level designation, the authors need to expand.
To align with and inform the large literature on growth cone biology, we adopted long-established standards to describe growth cone domains (e.g. Geraldo & Gordon-Weeks, PMID: 19812305). These standards are indeed macro level designations, in which the boundaries are approximate and dynamic. They are, nevertheless, based on cell morphology, an approach we could also use with low magnification EM overviews. One of the goals of our study was to shed more light on the functional significance of these field-adopted macro level designations by investigating the interplay of cytoskeletal ensembles at nanoscales. We have now added a relevant reference at the beginning of the second paragraph of the introduction to clarify that we use accepted domain nomenclature.

33. Also, why the Authors opt to acquire data exclusively on regions that are over the 2 µm diameter holes in the carbon. It will be informative to compare regions nearby on carbon associated with the same domains, as well as regions between domains while on carbon. Jasmin et al, 2016 suggested holes induce changes in the cytoskeleton of certain cells and the authors need to discuss and convince the reader that this is not an issue in the current study.
This is an interesting point. We chose to collect data over holes to reduce the background as much as possible in our data and to aid tilt-series alignment. The work of Jasnin et al (PMID: 27320835) investigated the behaviour of *Dictyostelium* cells on perforated supports, including cryo-EM grids, observing large-scale changes in the cytoskeleton at the circumference of these perforations. In our data, we saw no such perturbations. Two exemplar tomogram regions (displayed with 100 nm depth) are shown in Figure 3 for Reviewers , where the growth cone region stretches across the carbon substrate and a hole, with the edges of the hole (black arrows) and the overlying cytoskeleton networks displayed. Yellow arrowheads indicate examples of non-perturbed F-actin networks. (Scale bars = 100 nm).

© 2022. Published by The Company of Biologists under the terms of the Creative Commons Attribution License (https://creativecommons.org/licenses/by/4.0/).

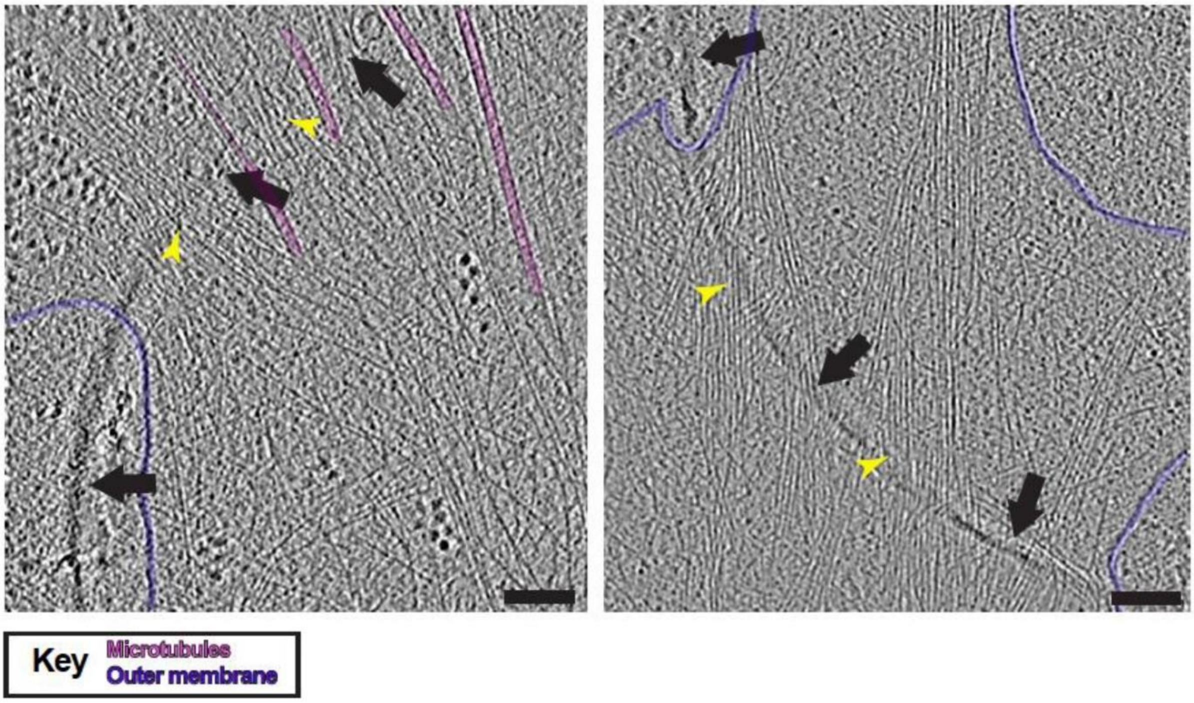

Figure 3 for Reviewers

The approach of visualising neuronal regions over cryo-EM grid holes and the non-perturbation of cytoskeleton features under investigation is consistent with other recent reports (e.g. Hoffman et al, 2021, PMID: 34698018; Foster et al (https://doi.org/10.1083/jcb.202103154). We therefore speculate that the phenomenon described by Jasnin et al is more cell-type specific, or at least not completely general; those authors' observations could also be related to the relatively minimal treatment of the perforated surface used, compared to our use of poly-lysine and laminin to treat the grid surfaces prior to neuron plating as described in the Methods (p18). Since i) the focus of our paper relates to growth cone biology, ii) there is precedent in the neuron literature for similar approaches and observations as ours, and iii) we are space-limited, we would propose not to introduce this discussion topic into our manuscript, but would be happy to follow editorial guidance on this point.

34. Concerning the effect of the missing wedge, the authors need to explain why they believe that the rotational symmetry of the holes ensures that their conclusions are "not significantly affected" by the potential attenuation of filament signals. The statement, as it stands, is unclear.
With apologies, we are not completely sure what the reviewer is referring to here and cannot find the phrase "not significantly affected" in our submitted manuscript. As described in other points (13 and 22), we acknowledge that we initially did not sufficiently account for the effects of the missing wedge, in particular in our analysis of the filopodia-derived actin bundles and have now addressed this (Fig. 3 and new Sup. Fig. 4). As we're sure the reviewer is aware, the sub-tomogram averaging we have performed e.g. on microtubules (Fig. 8B) eliminates missing wedge distortions.

35. The authors mentioned that neural network-driven density segmentation provided a quantitative analysis as well as absolute segmentation volumes were measured in Chimera. Can the authors expand on this topic? The validity of the quantification will depend on the quality of the NN segmentation, which, in turn, is not well defined and depends on how the user trains the network. To test and determine consistency, the authors should consider having at least three blinded and independent repeats of the NN segmentation and subsequent quantification by three different users and then report the statistics. Otherwise, the authors should remove any reference to "quantitative".
This is a fair point, and Reviewer 1 asked something similar (point 12). We have now expanded our description of the NN segmentation approach (p19) and included an additional illustrative Supplementary Figure (new Sup. Fig. 2). We use the resulting segmentation to determine the relative quantity of cellular components within each tomogram, and the combination of data from

© 2022. Published by The Company of Biologists under the terms of the Creative Commons Attribution License (https://creativecommons.org/licenses/by/4.0/).

multiple tomograms of equivalent regions of different growth cones allows statistical analysis, as already reported in Fig. 1A, B, F, H. We feel this is appropriately referred to as quantitative, and no other instances of “quantitative” analysis are present in the text.

36. Fascin: the authors are inclined to suggest that the architecture of P-domain actin bundles is most consistent with F-actin-Fascin crosslinks. This statement should be quantified, currently it is speculative and based on literature, not even measurements. Again, statistics of the measurements should be compiled and reported.
We agree with the reviewer that our data do not provide a direct demonstration that P-domain actin bundles are formed by fascin, and indeed the possibility of fascin’s involvement in these structures is only introduced in the Discussion. Here, and in response to a suggestion by reviewer 1 (point 14), we directly state that “filament dimensions, spacing and overall bundle architecture are most consistent with the involvement of fascin”. Comparison with the cited work of Claessens et al, which involved in vitro reconstitution of fascin-mediated bundles, is also consistent with this interpretation. These bundle properties are distinct from those formed by actin cross-linkers such as □-actinin (e.g. Hampton et al, 2007, PMID: 17331538) and espin (e.g. Claessens et al 2008, PMID: 18579789), and we have added a sentence to that effect in the Discussion text (p13).
Undertaking a statistically robust comparison of other possible actin cross-linkers as proposed by the reviewer would require collation and meta-analysis of a well-controlled set of experimentally equivalent data, which we consider to be beyond the scope of the current work. In summary, although we are clear about the limits of our data, we are convinced that there is good indirect evidence to support our suggestion that fascin is the major (although probably not sole) contributor to bundle formation.

37. Cofilin-bound actin filaments exist in the P-domain: Rigid-body fitted F-actin-cofilin exhibited a 0.87 model to map cross-correlation (CC) calculated in Chimera. The authors claim that this constitutes a “very good fit” is unconvincing. The value of the CC is highly sensitive to resolution, noise level and scaling and an absolute value is not meaningful. If the authors want to argue that it is more likely that these filaments contain cofilin than not, they need to conduct a comparative analysis where they compare fitting of bare actin (with adjusted pitch), fitting of actin with at least one alternative binding partner (eg dystrophin; again with adjusted pitch), and the fitting of actin-cofilin. This needs to be performed on at least three independent subsets of the data so statistics can be compiled. Then, the authors need to show that the statistics indicate actin-cofilin fits significantly better than bare actin and the alternative binder. Alternatively, the authors can remove the statement about the quality of the fit (including the value) and clarify in the text that their analysis is only qualitative and possible alternatives (including bare actin with modified twist) exist.
We agree with all the reviewer’s points. The purpose of our fitting analysis was to provide a qualitative comparison between the two F-actin populations we characterised. We have followed the reviewer’s suggestion and removed the statement about the quality of the fit. However, as illustrated in Sup. Video 4, the extra density present on the short-pitch filaments, together with prior information about the range of actin filament structures, suggests it is less likely that the short pitch filaments are bare actin with a modified twist.

38. Next the authors opt to capture tomograms within holes, can the authors quantify the ratio of observable filament detached from bundles when it does not occur at the hole edges? Holes, perforated surfaces were shown previously to affect actin nanoscale organization (Jasmin et al, 2016).
As noted in point 33 above, there is no evidence in our data of perturbations of growth cone actin cytoskeleton by the perforations in the cryo-EM grid surface

39. Arp2/3 mediated branches in the P-domain: Branching seems to be identified emanating from the sides of actin filaments, with ~ 70 degrees angels, were the angles measured, how many of them, and what were their SD?
These data were already in the manuscript, originally Fig. 3I, now Fig. 3G.

© 2022. Published by The Company of Biologists under the terms of the Creative Commons Attribution License (https://creativecommons.org/licenses/by/4.0/).

40. Close association of MTs with F-actin seems to be casual, as this observation is of key importance the authors, it needs to be quantified in 3D.
Our intention in presenting the data in Fig. 5G and H was to provide qualitative evidence of cytoskeleton filament proximity and we have adjusted the relevant text to reflect that.

Second decision letter

MS ID#: JOCES/2021/259234

MS TITLE: Visualising the cytoskeletal machinery in neuronal growth cones using cryo-electron tomography

AUTHORS: Joseph Atherton, Melissa Stouffer, Fiona Francis, and Carolyn A Moores
ARTICLE TYPE: Research Article

We have now reached a decision on the above manuscript.

To see the reviewers' reports and a copy of this decision letter, please go to: https://submit-jcs.biologists.org and click on the 'Manuscripts with Decisions' queue in the Author Area. (Corresponding author only has access to reviews.)

As you will see, the reviewers gave very favourable reports but raised few points that will require amendments to your manuscript. In addition, I strongly encourage you to submit your tomographic data sets to the most appropriate repository in order to allow the verification of the correctness of the averaged structure (validation report). I hope that you will be able to carry these out because I would like to be able to accept your paper, depending on further comments from reviewers.

We are aware that you may be experiencing disruption to the normal running of your lab that makes experimental revisions challenging. If it would be helpful, we encourage you to contact us to discuss your revision in greater detail. Please send us a point-by-point response indicating where you are able to address concerns raised (either experimentally or by changes to the text) and where you will not be able to do so within the normal timeframe of a revision. We will then provide further guidance. Please also note that we are happy to extend revision timeframes as necessary.

Please ensure that you clearly highlight all changes made in the revised manuscript. Please avoid using 'Tracked changes' in Word files as these are lost in PDF conversion.

I should be grateful if you would also provide a point-by-point response detailing how you have dealt with the points raised by the reviewers in the 'Response to Reviewers' box. Please attend to all of the reviewers' comments. If you do not agree with any of their criticisms or suggestions please explain clearly why this is so.

Reviewer 1

Advance summary and potential significance to field

./.

Comments for the author

Abstract. “Short” ended MTs has no well-defined meaning.Since there is a word limit, perhaps just say, “Microtubules”?
Legend of Fig. 2.While I admire the effort to be succinct, the description of the figures in 2A is so terse as to be incomprehensible.With work, I can figure out what is being said, but the legend is supposed to help the reader, not challenge him/her. I suggest making these descriptions more

© 2022. Published by The Company of Biologists under the terms of the Creative Commons Attribution License (https://creativecommons.org/licenses/by/4.0/).

readable by improving punctuation and added a few words, so not so much is left to the imagination.
Fig. 2D does a nice job of showing the hexagonal packing of the actin filaments and indicates the direction that will be affected by the missing wedge of data but nothing is said about why this matters. It is clear that each dark object
(filament cross-section) is longer in this direction and in the perpendicular, but this feature of the image is unexplained. A word about the impact of the missing data on shape would allow those who are not experts in cryoET to understand why the packing is hexagonal, but the actin filaments are asymmetric.

Reviewer 2

Advance summary and potential significance to field

Atherton et al. studied neuronal growth cone using cryo-electron tomography. The authors found some interesting aspects of cytoskeleton architecture organization that may be of interest to the community.

Comments for the author

I believe the reviewer answered my comments in a satisfactory manner and therefore I would have recommended to accept the manuscript for publication. However, the averaged structure were not submitted to the EMDB nor tomographic data sets. This is a common practice that allows to verify the correctness of the averaged structure (validation report).

Reviewer 3

Advance summary and potential significance to field

In the manuscript the authors aim at characterizing the mouse hippocampal neuron growth cones' 3D nanoarchitecture in its "central domains (C), transition zones (T) and peripheral domains (P) " as well as their interface with axons through the use of cryo-ET and machine learning-based segmentation . The authors employ semi manual segmentation analysis to assess the molecular distribution of cellular components within these different regions of WT growth cones and those of doublecortin Dcx knockout neurons (Dcx in mouse), a regulator of both growth cone F-actin and MT organization. The overall aim here is providing insights allowing unraveling the molecular basis for growth cone cytoskeleton regulation and coordination. A timely and exciting study.

Comments for the author

I appreciate the authors efforts and the manuscripts in its current form reads well.
Concerning point 35 in the response to reviewers , the authors mention they still consider their segmentation analysis "quantitative". Because the nature of the analysis is inherently subjective (it includes manual editing on top of specific training of NNs) I do not think this term is appropriate. As the authors refer to statistics when "quantitative" is mentioned, they should replace "quantitative" with "statistical".

© 2022. Published by The Company of Biologists under the terms of the Creative Commons Attribution License (https://creativecommons.org/licenses/by/4.0/).

Second revision

Author response to reviewers' comments

Reviewer 1 Comments for the Author:

1. Abstract. “Short” ended MTs has no well-defined meaning. Since there is a word limit, perhaps just say, “Microtubules”?
We had space to clarify meaning in the abstract and have adjusted the text accordingly.

2. Legend of Fig. 2.While I admire the effort to be succinct, the description of the figures in 2A is so terse as to be incomprehensible. With work, I can figure out what is being said, but the legend is supposed to help the reader, not challenge him/her. I suggest making these descriptions more readable by improving punctuation and added a few words, so not so much is left to the imagination.
We apologise for this over-compression of the text in the legend to Fig. 2. We have edited the text to improve reader comprehension while still keeping within the word limit.

3. Fig. 2D does a nice job of showing the hexagonal packing of the actin filaments and indicates the direction that will be affected by the missing wedge of data, but nothing is said about why this matters. It is clear that each dark object (filament cross-section) is longer in this direction and in the perpendicular, but this feature of the image is unexplained. A word about the impact of the missing data on shape would allow those who are not experts in cryoET to understand why the packing is hexagonal, but the actin filaments are asymmetric.
Thanks for this suggestion – we have added this information to the text at the bottom of p6.

Reviewer 2 Advance Summary and Potential Significance to Field:
Atherton et al. studied neuronal growth cone using cryo-electron tomography. The authors found some interesting aspects of cytoskeleton architecture organization that may be of interest to the community.

Reviewer 2 Comments for the Author:
4. I believe the reviewer answered my comments in a satisfactory manner and therefore I would have recommended to accept the manuscript for publication. However, the averaged structure were not submitted to the EMDB nor tomographic data sets. This is a common practice that allows to verify the correctness of the averaged structure (validation report).
As noted in our previous response (point 30), we are submitting exemplar tomograms from both WT and mutant neurons to EMDB and their deposition codes are now included in the Data Availability statement on p20. Similarly, we have also deposited the actin filament sub-tomogram reconstructions to EMDB and their deposition codes are also included in the Data Availability statement. The validation reports for the depositions are associated with each entry. We have further cut text throughout the rest of the manuscript to accommodate this extra section.

Reviewer 3 Advance Summary and Potential Significance to Field:
In the manuscript the authors aim at characterizing the mouse hippocampal neuron growth cones’ 3D nanoarchitecture in its “central domains (C), transition zones (T) and peripheral domains (P) “ as well as their interface with axons through the use of cryo-ET and machine learning-based segmentation . The authors employ semi manual segmentation analysis to assess the molecular distribution of cellular components within these different regions of WT growth cones and those of doublecortin Dcx knockout neurons (Dcx in mouse), a regulator of both growth cone F-actin and MT organization. The overall aim here is providing insights allowing unraveling the molecular basis for growth cone cytoskeleton regulation and coordination. A timely and exciting study.

Reviewer 3 Comments for the Author:
I appreciate the authors efforts and the manuscripts in its current form reads well.

5. Concerning point 35 in the response to reviewers , the authors mention they still consider their segmentation analysis "quantitative". Because the nature of the analysis is inherently subjective (it includes manual editing on top of specific training of NNs) I do not think this term is appropriate. As the authors refer to statistics when "quantitative" is mentioned, they should replace "quantitative"

© 2022. Published by The Company of Biologists under the terms of the Creative Commons Attribution License (https://creativecommons.org/licenses/by/4.0/).

with "statistical".
We respectfully disagree with the reviewer that “statistical” facilitates reader understanding in the contexts in which we previously used “quantitative”. To compromise, we have simply removed “quantitative” from the text (p5, p13, Legend to Fig.1, Fig. 5), and feel that the reader can still understand how the data were handled and what is being presented.

Third decision letter

MS ID#: JOCES/2021/259234

MS TITLE: Visualising the cytoskeletal machinery in neuronal growth cones using cryo-electron tomography

AUTHORS: Joseph Atherton, Melissa Stouffer, Fiona Francis, and Carolyn A Moores
ARTICLE TYPE: Research Article

I am happy to tell you that your manuscript has been accepted for publication in Journal of Cell Science, pending standard ethics checks.

© 2022. Published by The Company of Biologists under the terms of the Creative Commons Attribution License (https://creativecommons.org/licenses/by/4.0/).
